# Evolutionary history of Chaetognatha inferred from molecular and morphological data: a case study for body plan simplification

**DOI:** 10.1186/s12983-014-0084-7

**Published:** 2014-11-21

**Authors:** Samah Gasmi, Gabriel Nève, Nicolas Pech, Saïda Tekaya, André Gilles, Yvan Perez

**Affiliations:** Aix-Marseille Université, CNRS, IRD, Avignon Université, IMBE UMR 7263, 13331 Marseille cedex 3, France; Université de Tunis El Manar, Faculté des Sciences de Tunis, UR11ES12 Biologie de la Reproduction et du Développement animal, 2092 El Manar, Tunis Tunisie

**Keywords:** Chaetognatha, Phylogenetics, Systematics, Procrustes surimposition, Homoplasy, Body plan simplification

## Abstract

**Background:**

Chaetognatha are a phylum of marine carnivorous animals which includes more than 130 extant species. The internal systematics of this group have been intensively debated since it was discovered in the 18^th^ century. While they can be traced back to the earlier Cambrian, they are an extraordinarily homogeneous phylum at the morphological level - a fascinating characteristic that puzzled many a scientist who has tried to clarify their taxonomy. Recent studies which have attempted to reconstruct a phylogeny using molecular data have relied on single gene analyses and a somewhat restricted taxon sampling. Here, we present the first large scale phylogenetic study of Chaetognatha based on a combined analysis of nearly the complete ribosomal RNA (rRNA) genes. We use this analysis to infer the evolution of some morphological characters. This work includes 36 extant species, mainly obtained from *Tara Oceans* Expedition 2009/2012, that represent 16 genera and 6 of the 9 extant families.

**Results:**

Cladistic and phenetic analysis of morphological characters, geometric morphometrics and molecular small subunit (SSU rRNA) and large subunit (LSU rRNA) ribosomal genes phylogenies provided new insights into the relationships and the evolutionary history of Chaetognatha. We propose the following clade structure for the phylum: (((Sagittidae, Krohnittidae), Spadellidae), (Eukrohniidae, Heterokrohniidae)), with the Pterosagittidae included in the Sagittidae. The clade (Sagittidae, Krohnittidae) constitutes the monophyletic order of Aphragmophora. Molecular analyses showed that the Phragmophora are paraphyletic. The Ctenodontina/Flabellodontina and Syngonata/Chorismogonata hypotheses are invalidated on the basis of both morphological and molecular data. This new phylogeny also includes resurrected and modified genera within Sagittidae.

**Conclusions:**

The distribution of some morphological characters traditionally used in systematics and for species diagnosis suggests that the diversity in Chaetognatha was produced through a process of mosaic evolution. Moreover, chaetognaths have mostly evolved by simplification of their body plan and their history shows numerous convergent events of losses and reversions. The main morphological novelty observed is the acquisition of a second pair of lateral fins in Sagittidae, which represents an adaptation to the holoplanktonic niche.

**Electronic supplementary material:**

The online version of this article (doi:10.1186/s12983-014-0084-7) contains supplementary material, which is available to authorized users.

## Background

Chaetognaths are small predators of major importance in the marine ecosystem [1,2]. They are abundant in every sea worldwide and can be traced back to the Cambrian radiation [3]. Most of them are planktonic but a few are benthic. Chaetognaths are particularly renowned for their peculiar morphological and developmental features. These characters, as well as the affinities of the group within the metazoans, have been extensively debated by zoologists since the discovery of the phylum in the 18th century [4]. So far the most recent phylogenetic analyses have also proved problematic for inferring their sister-group relationships within metazoans, which makes their positioning one of the most difficult issues in animal phylogeny [5]. Numerous alternative phylogenetic hypotheses have been proposed over a long history of debate (for review [5,6]). However, a recent hypothesis has emerged, based on morphological [7,8] and phylogenomic analyses [9-13], where chaetognaths have been considered an early diverging member of Protostomia. The circumoral brain and the intraepithelial ventral cords have been recognized to be two of the key apomorphies of Protostomia [14,15]. The nervous system in Chaetognatha is characterized by such a typical arrangement. However, even though the Chaetognatha partly share the Protostomia ground pattern, Perez et al. [5] concluded that “their derived genome and morphology do not include any convincing synapomorphy that would suggest a sister-group relationship to another metazoan taxon”.

**Figure 1:**
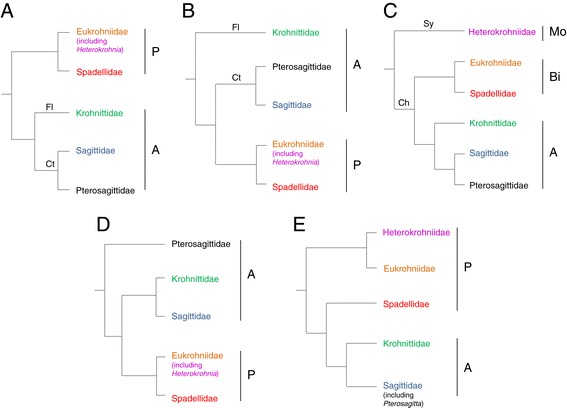
**Five main phylogenetic hypotheses of chaetognaths relationships.** Hypothesis based on morphological data: A, Tokioka [21]. **B**, Tokioka [22], **C**, Casanova [24], **D** Salvini-Plawen [26]. **E**, data presented here. A, Aphragmophora; Bi, Biphragmophora; Ch, Chorismogonata; Ct, Ctenodontina; Fl, Flabellodontina; Mo, Monophragmophora; P, Phragmophora; Sy, Syngonata.

As their relationships within metazoans, their internal systematics is still very much debated [16-18]. Here, we recall the main hypotheses previously proposed based on morphological and, more recently, molecular data. According to Ritter-Zahony [19] and Hyman [20], Chaetognatha was traditionally divided into six genera representing four families: *Sagitta* (Sagittidae), *Pterosagitta* (Pterosagittidae), *Spadella*, *Eukrohnia* and *Heterokrohnia* (Eukrohniidae) and *Krohnitta* (Krohnittidae). Later, Tokioka [21] re-evaluated the relationships between families by creating two new orders (Figure 1A): the plesiomorphic Phragmophora (presence of a transverse musculature, namely the phragms, and various kinds of glandular structures on the body surface) composed of Spadellidae and Eukrohniidae; and the derived Aphragmophora (absence of phragms and few glandular structures). Tokioka [21] suggested creating two Aphragmophora suborders according to the shape of teeth and hooks and the number of teeth rows. The suborder Flabellodontina only contains the family Krohnittidae, while the Pterosagittidae and Sagittidae belonged to the Ctenodontina. In a following work, Tokioka [22] suggested the paraphyly of Aphragmophora (Figure 1B), with the Ctenodontina were thought to be closer to the Phragmophora than to the Flabellodontina. Inspired by a previous suggestion of Alvariño [23], Tokioka [21] considered that some of the structural differences between *Sagitta* species were of significant systematic importance. This author divided *Sagitta* into nine new genera and gathered them into the Sagittidae. After the discovery of several new deep benthoplanktonic chaetognaths, Casanova [24] slightly modified Tokioka’s hypothesis (Figure 1C). In his version, the members of the Phragmophora order were split into two new orders. First, the Biphragmophora (comprising the new Heterokrohniidae family, with transverse muscles in both trunk and tail) was included into the subclass Syngonata (with ducts between the genital glands). Second, the Monophragmophora (Spadellidae and Eukrohniidae, with transverse muscles in trunk only) was associated with the Aphragmophora into the subclass Chorismogonata (without such ducts). Using multivariate analyses based on body shape, Dallot and Ibanez [25] suggested the existence of three groups (*Sagitta*, *Eukrohnia*, and *Spadella*/*Bathyspadella*) and a close relationship between the planktonic species *Pterosagitta draco* and the benthic Spadellidae. They also questioned the inclusion of *Sagitta lyra* within the genus *Sagitta*. In another study, Salvini-Plawen [26] proposed the littoral-neritic *Pterosagitta draco* as the most plesiomorphic species and contradicted the ancestrality of phragms (Figure 1D). He also omitted the Aphragmophora suborders Ctenodontina and Flabellodontina of Tokioka [21] and the Syngonata/Chorismogonata hypothesis of Casanova [24]. Finally, Bieri [21,22] proposed the most recent revision of the chaetognaths classification. Following Alvariño [23] and Tokioka [21,22], he suggested new genera within Sagittidae. Several morphological criteria were taken into account: position and shape of the corona ciliata; position and shape of lateral fins and seminal vesicles; presence/absence and shape of the intestinal diverticula; trunk/tail length ratio; rayless-zones in the lateral fins; body aspect. This author also disregarded the Syngonata/Chorismogonata hypothesis.

Thus, based on a consensus between Tokioka, Casanova and Bieri’s hypotheses, the extant Chaetognatha are represented by three orders (Biphragmophora, Monophragmophora, Aphragmophora) and nine families (Heterokrohniidae, Eukrohniidae, Pterokrohniidae, Spadellidae, Krohnittellidae, Krohnittidae, Pterosagittidae, Sagittidae, Bathybelosidae).

The first molecular study of chaetognaths systematics was conducted with a short portion of the large subunit ribosomal RNA 28S (LSU rRNA) gene [27]. These authors concluded that the LSU rRNA gene is duplicated in Chaetognatha, the division into Aphragmophora and Phragmophora is supported and several genera of the Sagittidae family described by Tokioka [21] and Bieri [16] are recovered. Papillon et al. [18] carried out a more extensive molecular study based on 26 sequences of the small subunit ribosomal RNA 18S (SSU rRNA) isolated from members of six extant families; they concluded that (1) similarly to LSU rRNA, a duplication of SSU rRNA gene occurred, suggesting that the whole ribosomal cluster is duplicated, (2) Tokioka’s suborders Ctenodontina and Flabellodontina are not validated, (3) Casanova’s hypotheses Syngonata/Chorismogonata and Monophragmophora/Biphragmophora are rejected, (4) the families Krohnittidae and Pterosagittidae are not supported, (5) three monophyletic groups are identified: Sagittidae/Krohnittidae, Spadellidae/Pterosagittidae and Eukrohniidae/Heterokrohniidae, (6) the order Aphragmophora without *Pterosagitta draco* is monophyletic. Since then, no molecular study has been made to further explore the systematics of this phylum. Finally, a recent barcoding analysis was highly successful at discriminating between the described species [28]. It notably revealed little geographical structure and showed that *Eukrohnia bathypelagica* and *Eukrohnia hamata* are probably young sister-species.

Thus, even after one century of heavy debates, it has not been possible to establish a stable and reliable hypothesis on the evolutionary history of Chaetognatha. In the present work, we have conducted an extensive molecular analysis based on LSU and SSU rRNA duplicated genes. We combined, for the first time, the molecular results with a morphological classification and geometric morphometrics. In the light of our results, we present a revised phylogeny and discuss the morphology-based character systems that have traditionally been used to classify this enigmatic phylum.

## Results

### Alignments and erroneous sequences

First, we identified erroneous sequences by constructing test trees from LSU and SSU rRNA genes and by then identifying which sequences from public databases came out in suspicious positions when compared with new sequences obtain in the present study. This approach revealed three chaetognath sequences that are erroneous due to contaminant or bad species diagnosis. These sequences that must be excluded from any future phylogenetic analyses were:

- The SSU rRNA of *Krohnitta pacifica* (class I DQ351879 and class II DQ351891) from [18] which most likely is a Sagittidae contaminant close to *Parasagitta setosa* when compared with three new sequences belonging to *Krohnitta subtilis*.
- The SSU rRNA of *Sagitta sp* (class I AY922316) from [29] which belongs to the Eukrohniidae family, close to *Eukrohnia hamata* and *Eukrohnia bathypelagica*.
- We also characterised new sequences for three different specimens of *Pterosagitta draco* that were used instead of the erroneous SSU rRNA sequences (class I DQ351885 and class II DQ351898) from [18].

After excluding these contaminants, we set up five alignments available upon request (dataset 1, LSU rRNA paralogous genes; dataset 2, SSU rRNA paralogous genes; dataset 3, SSU rRNA paralogy class I; dataset 4, SSU rRNA paralogy class II; dataset 5, concatenated alignment of SSU and LSU rRNA genes).

### Molecular phylogenetic analyses

Whatever the dataset considered, the Bayesian and approximate likelihood ratio (aLRT) trees obtained were almost identical to the maximum likelihood (ML) bootstrapped tree. Thus their statistical values were reported on the ML topology. However, in the case of the concatenated analysis, because only the aLRT and the Bayesian reconstructions showed a fully congruent topology, statistical values obtained from the three methods were reported on the Bayesian topology. The validity of the Krohnittidae and Heterokrohniidae families will not be discussed because both are only represented here by a single species.

**Figure 2:**
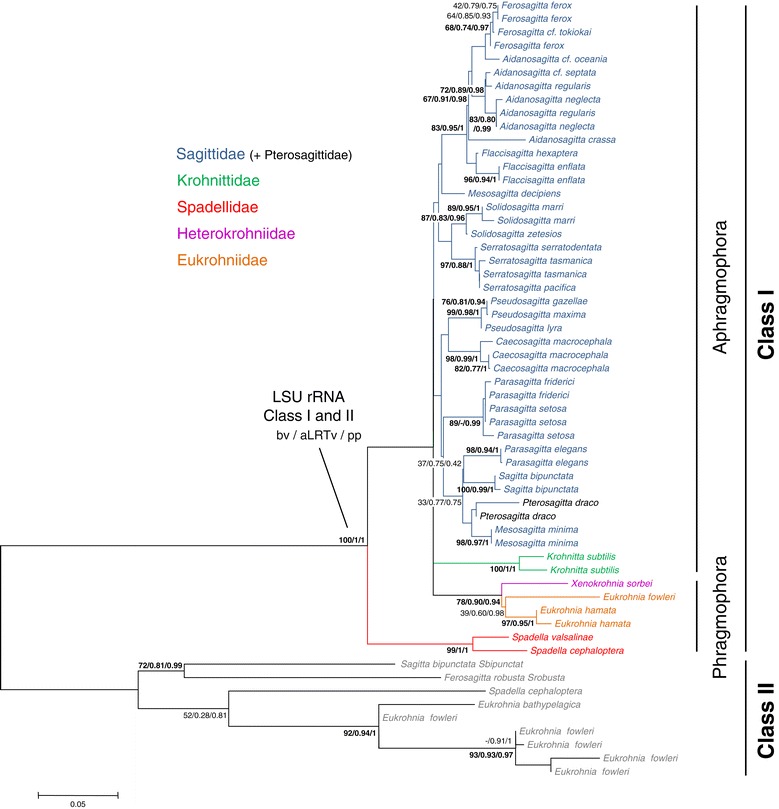
**Maximum likelihood tree calculated from LSU rRNA by the Mega program with the K2P + Γ model.** The alignment of 58 sequences of LSU rRNA was 410 base pairs long. The lnL value of this optimal tree is −2457.20. The two classes of LSU rRNA yielded trees that rooted each other. Support values obtained using different reconstruction approaches are indicated at nodes in the following order: maximum Likelihood bootstrap probabilities (bv), approximative likelihood ratio test (aLRTv), and Bayesian posterior probabilities (pp). Support values are displayed when bv/aLRTv ≥75 or pp ≥0.85. Node absence in a given method is indicated by -.

*Large subunit rRNA*. The alignment of 58 sequences of LSU rRNA (49 specimens for the class I and 9 for the class II) was 410 base pairs (bp) long (dataset 1 = LSU rRNA Class I and II). The best model of evolution estimated with MODELTEST was the K2P + Γ model (with a lnL of −2457.20). Without the nine class II sequences, the same model was selected (with a lnL of −1483.09). Using the midpoint rooting, the two classes of LSU rRNA yielded two monophyletic groups (Figure 2). The rate of substitution for the unconstrained tree was equal to 8.16 × 10^−2^ ([8.02 × 10^−2^ – 8.30 × 10^−2^] 95% confidence interval). Only the topology based on class I sequences is discussed. Parametric (approximate likelihood ratio values, aLRTv and posterior probabilities, pp) and non-parametric branch tests (bootstrap values, bv) showed a recurrent lack of resolution for the deep nodes of trees based on LSU rRNA genes. To assess whether a consistent phylogenetic signal could be revealed by reducing the impact of noise, the same phylogenetic analyses were carried out, but this time after treatment of the dataset 1 with the program Aliscore. The new LSU rRNA alignment (Dataset 1bis) displayed 155 (37.8%) ‘noisy’ sites that were filtered out and did not impact on the selected model of evolution (K2P + Γ with a lnL of −1735.6). Most of these potential ‘noisy’ sites were located in the loop regions of the LSU rRNA gene class II due to *Xenokrohnia sorbei* LSU rRNA gene class I alignment. After removing these positions, the accuracy of the trees did not increase (data not shown). Thus, the lack of resolution for deep branching nodes is more likely linked to a lack of phylogenetic information than to a ‘noisy’ signal due to mismatches in sequence alignment (*i.e.*, soft polytomy). Because of this, relationships between orders and families were very difficult to assess. LSU rRNA sequences contained information only relevant to the discussion of the relationships between species of a given genus and, to a lesser extent, between genera of the same family. However, the trees constructed under ML and Bayesian criteria produced very similar topologies if considering only the well-resolved nodes. First, Aphragmophora (Krohnittidae + Sagittidae + Pterosagittidae) and Phragmophora (Heterokrohniidae + Eukrohniidae + Spadellidae) were not monophyletic. A close relationship between the Eukrohniidae and *X. sorbei*, the only representative of the Heterokrohniidae, was well supported in all methods (78/0.90/0.94, bv/aLRTv/pp hereinafter). At the family level, only the Spadellidae received high support whatever the method considered (99/1/1). The monophyly of Eukrohniidae was supported only in the Bayesian analysis (39/0.60/0.98). The monophyly of the Sagittidae, which was the most represented family (25 species/11 genera), cannot be confirmed because of very low support value in ML analysis (bv = 20) or the lack of node in aLRT and Bayesian analyses. It is however possible to characterize two main lineages within this family. Within Sagittidae, a well-supported assemblage consisted of *Ferosagitta*, *Aidanosagitta* and *Flaccisagitta* (83/0.95/1). A surprising result was the inclusion of *Pterosagitta draco*, the only representative of the Pterosagittidae, within Sagittidae, close to *Sagitta*, *Parasagitta* genera and *Mesosagitta minima*, but with weak support (33/0.77/0.75). Several genera validated in the Bieri’s [16] nomenclature appeared monophyletic with marginal or high support values. These are *Ferosagitta* (two of the seven known species are represented, 68/0.74/0.97), *Solidosagitta* (two of the three known species are represented, 87/0.83/0.96), *Serratosagitta* (three of the five known species are represented, 97/0.88/1), *Pseudosagitta* (three of the four known species are represented, 99/0.98/1). Yet *Flaccisagitta* (two of the three known species are represented) received low support values from the ML analysis and marginal support values from Bayesian analysis (38/0.84 respectively) whereas this node was not recovered in the aLRT tree. The remaining genera are either monospecific (*Caecosagitta*, *Pterosagitta*) or did not appear monophyletic but were grouped in tight assemblages. It is especially the case of large and heterogeneous genera such as *Aidanosagitta* and *Parasagitta*. The *Mesosagitta* genus, represented here by *M. minima* and *M. decipiens*, was invalidated.

*Small subunit rRNA*. Three data sets of SSU rRNA sequences were analysed. First, phylogenetic reconstructions were conducted with dataset 2 comprising all available SSU rRNA sequences from both paralogy classes (dataset 2 = SSU rRNA Class I and II, 138 sequences from 33 species: 80 class I and 58 class II sequences). One of the recurrent problems when analysing the molecular data in Chaetognatha is the lack of relevant outgroups among bilaterians. So, this first analysis offered to root one paralogy class on the other, highlighting the branching order of five represented families with good statistical values and revealing the following sequence from the last common ancestor to the most derived family (Additional file 1): Eukrohniidae + Heterokrohniidae, Spadellidae, Krohnittidae and Sagittidae, the latter including Pterosagittidae. Similarly to the LSU rRNA analysis, Phragmophora (Heterokrohniidae + Eukrohniidae + Spadellidae) were paraphyletic while Aphragmophora (Pterosagittidae + Sagittidae + Krohnittidae) received high support in most methods applied (class I 80/1/0.98, class II 19/1/0.85).

**Figure 3:**
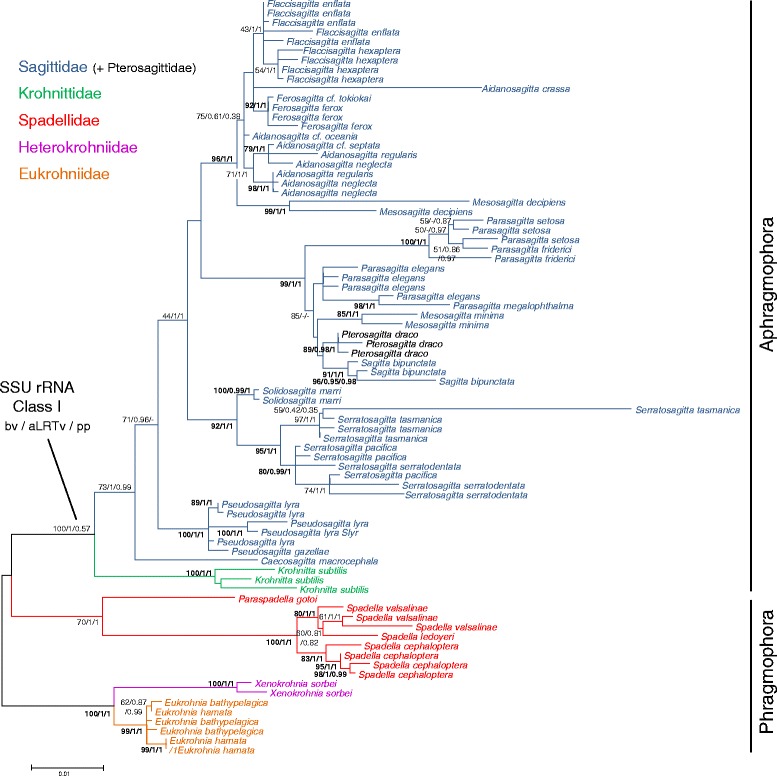
**Maximum likelihood tree calculated from SSU rRNA Class I by the Mega program with the K2P + Γ model.** The alignment of 80 sequences of LSU rRNA was 1679 base pairs long. The lnL value of this optimal tree was −7733.81. Support values obtained using different reconstruction approaches are indicated at nodes in the following order: maximum Likelihood bootstrap probabilities (bv), approximative likelihood ratio test (aLRTv) and Bayesian posterior probabilities (pp). Support values are displayed when bv/aLRTv ≥75 or pp ≥0.85. Node absence in a given method is indicated by -. Tree was rooted on the monophyletic assemblage consisting of Eukrohniidae + Heterokrohniidae. This rooting was also observed in most analyses when using the midpoint rooting function.

**Figure 4:**
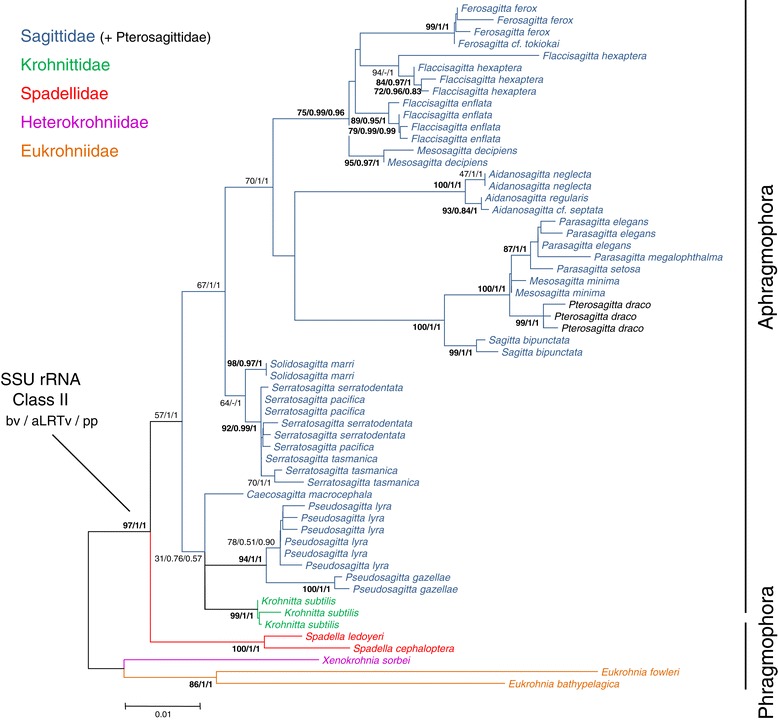
**Maximum likelihood tree calculated from SSU rRNA Class II by the Mega program with the T92 + Γ model.** The alignment of 58 sequences of LSU rRNA was 1119 base pairs long. The lnL value of this optimal tree was −4073.31. Support values obtained using different reconstruction approaches are indicated at nodes in the following order: maximum Likelihood bootstrap probabilities (bv), approximative likelihood ratio test (aLRTv) and Bayesian posterior probabilities (pp). Support values are displayed when bv/aLRTv ≥75 or pp ≥0.85. Node absence in a given method is indicated by -. Tree was rooted on the monophyletic assemblage consisting of Eukrohniidae + Heterokrohniidae. This rooting was also observed in most analyses when using the midpoint rooting function.

Datasets 3 (SSU rRNA Class I, 1679 bp long) and 4 (SSU rRNA Class II, 1119 bp long) consisted of two independent alignments for each paralogy. Here, we only present the analyses based on these two datasets (Figures 3 and 4 respectively) because separate phylogenetic reconstructions based on one paralogous gene gave more robust and accurate results than the full data set comprising both SSU rRNA paralogy classes (dataset 2). The selected model was the K2P + Γ with a lnL of −7733.81 for the class I and the T92 + Γ with a lnL of −4073.31 for the class II. The substitution rates were equal to 1.14 × 10^−2^ ([4.19 × 10^−4^ – 2.30 × 10^−2^] 95% confidence interval) for the SSU rRNA class I and 1.03 × 10^−2^ ([4.83 × 10^−4^ – 2.14 × 10^−2^] 95% confidence interval) for the SSU rRNA class II. When looking at the overall alignments based on both SSU rRNA paralogy classes, they included 579 variable sites (34.48%). This number decreased to 446 in the SSU rRNA class I (26.56%) and to 252 in the SSU rRNA class II (22.52%). However, the number of highly supported nodes within the ML phylogenies (bv >75) increases from 44 when analysing both SSU rRNA classes together to 56 (32 in class I and 24 in class II) when analysing each class separately. In fact, the evolutionary rates of the two paralogous genes are very close to each other. However, while transitions and tranversions are each responsible for 50% of the substitutions in class I, transitions are involved for 68% in class II. This explains the selection of two different evolutionary models by MODELTEST. Therefore, the difference in distance estimation between the classes I and II is more likely due to the lower frequency of transversion events in class II than to base composition. According to the topology obtained in the analyses of both classes of SSU rRNA genes (see Additional file 1), separate analyses of each paralogy classes were rooted on the monophyletic assemblage consisting of Eukrohniidae + Heterokrohniidae. This rooting was also observed in most analyses when using the midpoint rooting function. The overall topologies obtained with SSU rRNA sequences belonging to class I and II (Figures 3 and 4 respectively) were more robust than with the LSU rRNA sequences. The monophyletic group consisting of *X. sorbei* and all species belonging to Eukrohniidae was unambiguously supported in all methods applied (class I 100/1/1, class II 97/1/1 hereinafter). As with the LSU analysis, Aphragmophora (Krohnittidae + Sagittidae + Pterosagittidae) received good support (100/1/0.57, 57/1/1). All the families (Eukrohniidae 99/1/1, 86/1/1; Spadellidae 70/1/1, 100/1/1; Sagittidae + Pterosagittidae 73/1/0.99, class I only) were highly supported by SSU trees. In all methods used, the branching order of these clades was extremely well conserved between both paralogy classes, with one main difference being the position of Krohnittidae. This monogeneric family comprising three species (but only represented here by *Krohnitta subtilis*) was found either as the sister-group to Sagittidae in the class I trees with high support (100/1/0.57) or within Sagittidae (57/1/1) in class II trees in a mongrel assemblage comprising *Caecosagitta* and *Pseudosagitta* genera but with low support (31/0.76/0.57). Several traditional genera were not monophyletic, for instance *Mesosagitta* (*M. minima* and *M. decipiens*) which was invalidated by our results. *Aidanosagitta* was paraphyletic in class I analysis and monophyletic with high support in class II analysis (100/1/1) while the opposite was obtained for *Flaccisagitta*, the monophyly of which received marginal bv and high aLRTv and pp values in class I analysis (43/1/1). *Parasagitta* was paraphyletic in the class I analysis and monophyletic in the class II analysis with high support (87/1/1). Three main subclusters were identified within Sagittidae. *Aidanosagitta*, *Flaccisagitta*, two *Ferosagitta* species (*F. tokiokai* and *F. ferox*) and *Mesosagitta decipiens* formed a first clade in the class I trees with high support (96/1/1). In class II trees, only *Flaccisagitta, Ferosagitta and M. decipiens* were clustered together with high support (75/0.99/0.96). *Parasagitta*, *P. draco*, *Sagitta bipunctata* and *M. minima* constituted a second subcluster with high support in all trees (99/1/1, 100/1/1). This second subcluster displayed a close relationship with the first one in the class II analysis (70/1/1). A third and last Sagittidae subcluster consisting of *Solidosagitta* and *Serratosagitta* genera was also found to be monophyletic but was highly supported only in class I analyses (92/1/1, 64/-/1). Finally, the class I analysis confirmed the monophyly of *Pseudosagitta* (*P. lyra* and *P. gazellae*) with the three main Sagittidae subclusters only in the ML and aLRT trees (71/0.96/-) and the most basal genus among all Sagittidae was the monospecific genus *Caecosagitta* (73/1/0.99). The trees resolution drastically decreased at the infrageneric level and phylogenies based on SSU rRNA data did not recover the monophyly of several species, for instance in *Serratosagitta pacifica* and *S. serratodentata* (class I and II), *M. minima* (class II), *Aidanosagitta regularis and A. neglecta* (class I), *Parasagitta elegans* (class I and II), *Parasagitta setosa and Parasagitta friderici* (Class I), *P. lyra* (class I), *F. ferox* (class I), *Eukrohnia hamata* and *E. Bathypelagica* (class I).

*Concatenated analysis.* As it is done for large coding genes that contain considerable phylogenetic signal, rRNA genes were concatenated to increase the accuracy of the phylogenetic reconstructions (Dataset 5 = SSU rRNA Class I, SSU rRNA Class II and LSU rRNA Class I concatenated). The total length of the concatenation based on the two paralogous SSU rRNA class I and class II (1670 and 1087 pb respectively) and LSU rRNA class I (366 pb) was 3123 bp. We conserved as many taxa as possible in the concatenated approach, *e.g.*, all species showing at least two genes and two specimens. *Eukrohnia bathypelagica* and *Paraspadella gotoi* displayed SSU rRNA sequences only for class I and *Eukrohnia fowleri* only for class II. The model of evolution was estimated on the full concatenated data set without any partitioning of the data. The selected model was the GTR + Γ + I with a lnL of −15473.70 with the dataset comprising 55 sequences and lnL of −13443.12 with the dataset comprising 53 sequences.

**Figure 5:**
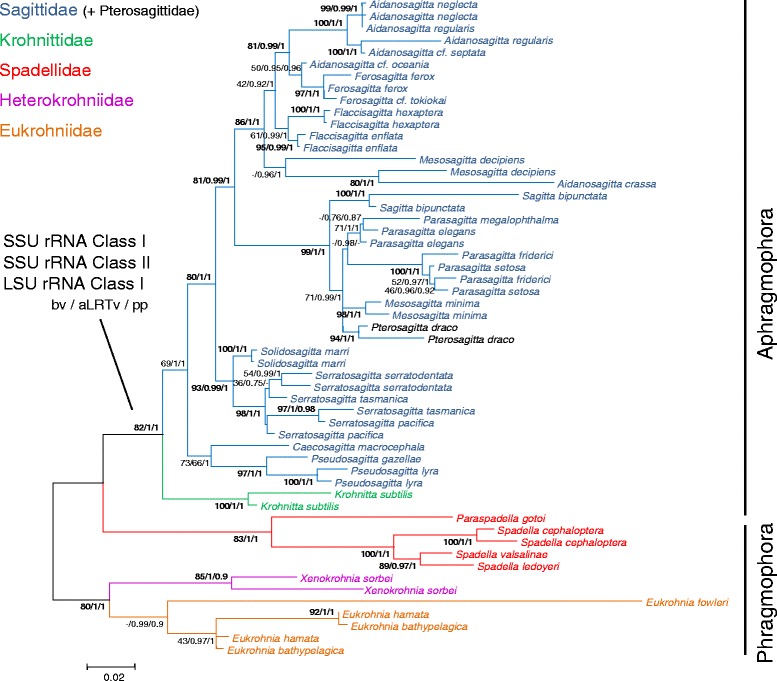
**Bayesian majority-rule consensus tree obtained from a supermatrix including both paralogy classes of SSU rRNA and LSU rRNA class I genes under the GTR + Γ + I model.** The alignment of 55 concatenated rRNA sequences was 3123 base pairs long. Support values obtained using different reconstruction approaches are indicated at nodes in the following order: maximum Likelihood bootstrap probabilities (bv), approximative likelihood ratio test (aLRTv) and Bayesian posterior probabilities (pp). Support values are displayed when bv/aLRTv ≥75 or pp ≥0.85. Node absence in a given method is indicated by -. Tree was rooted using the midpoint rooting function.

Trees resulting from concatenation (Figure 5) showed a very similar topology to the one obtained from the SSU rRNA genes and, to a certain extent, from the LSU rRNA genes, with a better accuracy not only at the family rank but also at the sub family and genus ranks. ML, aLRT and Bayesian analyses rooted on midpoint were consistent with the topology obtained in the rooted analysis including both SSU rRNA classes of paralogy and yielded the monophyly of the Aphragmophora (82/1/1) and paraphyly of the Phragmophora. Eukrohniidae, Spadellidae and Sagittidae families were monophyletic with high supports in most methods employed (−/0.99/0.90, 83/1/1 and 69/1/1 respectively). Heterokrohniidae was sister group to Eukrohniidae (80/1/1) and Krohnittidae was sister group to Sagittidae (82/1/1). Topologies based on the larger matrix were also well resolved for relationships within families and were similar to the ones obtained from single gene rRNA analyses. Specifically, within Eukrohniidae, *Eukrohnia fowleri* appeared basal with aLRTv and Bayesian posteriors (−/0.99/0.90) and *E. hamata* and *E. bathypelagica* showed close relationships (43/0.97/1). Moreover, these latter two species were paraphyletic to each other. *Paraspadella* was basal within Spadellidae (83/1/1). Within *Spadella* (100/1/1), *S. valsalinae* was found to be sister to *S. ledoyeri* (89/0.97/1). Finally, within the Sagittidae, the clade *Caecosagitta + Pseudosagitta* was supported with high Bayesian posteriors only (73/66/1) and appeared sister to all other Sagittidae species. As in the single gene analyses, three main lineages of Sagittidae were recovered with high supports. These were: *Solidosagitta* + *Serratosagitta* (93/0.99/1), *Parasagitta* (including *M. minima*) + *Sagitta* + *P. draco* (99/1/1) and *Flaccisagitta* + *Aidanosagitta* + *Ferosagitta* + *M. decipiens* (86/1/1). As with the separate analysis of SSU rRNA genes, the resolution of the relationships within Sagittidae decreased at the infrageneric level and the monophyly of several species was not supported. This was the case for the following species: *S. tasmanica and S. pacifica*, *M. decipiens*, *A. neglecta, P. elegans, P. setosa and P. friderici*, *E. hamata and E. bathypelagica*.

### Cladistic and phenetic analysis by morphology

**Figure 6:**
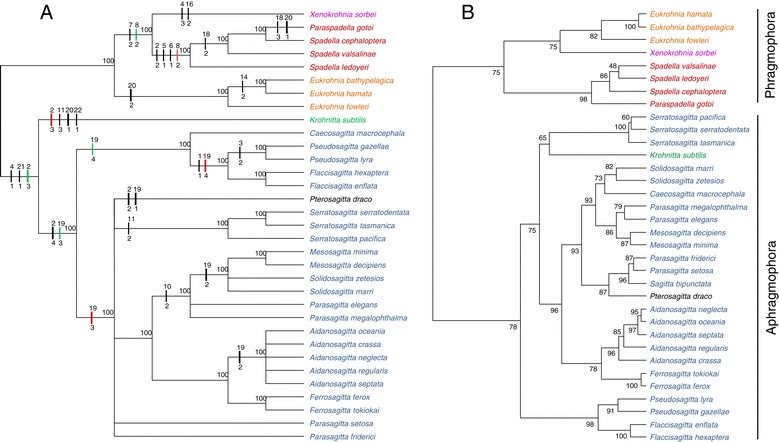
**Cladistic and phenetic analyses of morphological data. A**: Cladistical analysis of morphological data based on 23 qualitative characters. Majority rule consensus tree of 340 equally parsimonious tree (consistency index = 0.633; retention index = 0.837) obtained with the exclusion of *Sagitta bipunctata*. Majority rule consensus values are shown above internal branches. Only synapomorphies presenting a consistency index = 0.6 are shown on the branches. Black bars represent synapomorphies with the corresponding morphological character number above and the character state change below. Red and Green bars correspond to alternative hypotheses according to DELTRAN or ACCTRAN optimisation respectively. Characters coding is presented in Additional file 2. **B**: Phenetic analysis of morphological data based on 23 qualitative and 9 quantitative characters. Approximately unbiased test values are shown above internal branches. Characters coding is presented in Additional file 2.

Because it is impossible to choose an appropriate outgroup to root a cladistic analysis of chaetognaths morphological features, we performed a first analysis using the MinF option that minimizes the f-value [30]. Among the 23 characters, 21 were cladistically informative. The Heuristic MP search found 12,435 trees 65 steps long. The consistency index (CI, excluding uninformative characters) was equal to 0.5846 with a retention index (RI) of 0.8043, a homoplasy index (HI) of 0.4154 and a g1 of −0.4702. The CI excluding uninformative characters was equal to 0.5714. When we removed species with identical morphological coding (9 taxa, 26.47% of the data set) 20 characters were informative. We found 4,115 trees showing a CI of 0.603, a RI of 0.779, a HI of 0.397, and a g1 of −0.706. As identical morphological sequences had no impact on tree topology, we analysed the complete data set. The consensus topology rooted on midpoint yielded Phragmophora and Aphragmophora monophyletic (data not shown). On the basis of this topology, we conducted two constraint analyses rooted on Phragmophora for character-state optimization under ACCTRAN and DELTRAN settings (Figure 6A). In the following, we will only discuss the synapomorphies with a consistency index equal or higher than 0.6. Within Phragmophora, the relationships were well resolved with the monophyly of the Eukrohniidae (100, character #20 one row of posterior teeth) and the monophyly of *Xenokrohnia sorbei* + Spadellidae (100, character #7 heterosarcomeric secondary muscle). Within the latter, *Spadella* was paraphyletic with *Paraspadella gotoi* as the most derived genus of Spadellidae. The optimization of the character #8 changed when ACCTRAN setting was selected and appeared as a synapomorphy of the grouping *X. sorbei* + Spadellidae while it was a synapomorphy of the Spadellidae only when DELTRAN was selected. This slight difference between ACCTRAN and DELTRAN optimization is likely due to the lack of data for character #8 in *X. sorbei* (see Additional file 2). Four synapomorphies were found with DELTRAN setting for the Spadellidae (100, characters #2 one short pair of lateral fins, the anterior end at the level of the caudal septum, #5 phragms with supercontraction, #6 longitudinal muscle with B fibres only, #8 organisation of RFamide-like neurons type B). We identified one species, *Sagitta bipunctata*, responsible for a decrease of the branching resolution within Aphragmophora (data not shown). When the analysis was conducted with *S. bipunctata*, *Krohnitta subtilis* and *Caecosagitta macrocephala* split first and constituted two distinct lineages. The next cladogenesis event received moderate support (70) and yielded a group consisting of *Pterosagitta draco* and the remaining Sagittidae were left in a broad polytomy. Nevertheless, three groupings received good or moderate support for instance *Flaccisagitta* with *Pseudosagitta* (100 characters #1 flaccid body), *Aidanosagitta* with *Ferosagitta* (95) and *Mesosagitta* with *Solidosagitta* (76). We found that characters #9 (intestinal diverticula) and # 13 (seminal vesicles close to tail fin and well separated from lateral ones) were primarily responsible for the HI increase. When removing *S. bipunctata* from the analysis, the number of resulting trees decreased to 340 (62 steps) showing a CI of 0.6129, a RI of 0.8222, a HI of 0.3871 and a g1 of −0.5039. We found three synapomorphies for Aphragmophora (100) under the ACCTRAN optimization (characters #2 one pair of lateral fins with the anterior end and the posterior end at equal distance from the caudal septum, #4 absence of phragms and #21 glandular structures on the body surface scarcely developed). Nevertheless, this state of character #2 appeared to be an autapomorphy of *K. subtilis* with DELTRAN settings. Sagittidae including *P. draco* were monophyletic with high support (100). This clade exhibited two synapomorphies under the ACCTRAN optimization (characters #2 two pairs of lateral fins, #19 corona ciliata type C) while one of which (characters #19) was synapomorphic to *C. macrocephala*, *Flaccisagitta* and *Pseudosagitta* under DELTRAN settings. The sister-group relationship between *Flaccisagitta and Pseudosagitta* was confirmed (100, characters #1 flaccid body) with *C. macrocephala* basal to this grouping (100). The next cladogenesis event grouped *P. draco* with the remaining Sagittidae (100), with character #19 as synapomorphy with DELTRAN settings. These differences between ACCTRAN and DELTRAN optimisations were a consequence of a lack of description of the corona ciliata in *C. macrocephala* (see Additional file 2). Once again the overall relationships between Sagittidae genera were not fully resolved, except for the sister-group relationships between *Ferosagitta* + *Aidanosagitta* (100, no synapomorphy found) and *Parasagitta* + *Solidosagitta* + *Mesosagitta* (100, character #10 vacuolated intestinal cells). *Serratosagitta* (100, character #11 serrated hooks) as well as *Aidanosagitta* were monophyletic (100) but the latter was defined by a convergence with *Mesosagitta* and *Solidosagitta* (character #19 corona ciliata type B). *Parasagitta* and *Solidosagitta* were not monophyletic. Finally, *P. draco* was characterized by two homoplasic states in respect to Spadellidae due to the reversion of the characters #2 (one short pair of lateral fins, the anterior end at the level of the caudal septum) and #19 (corona ciliata type A).

We conducted an alternative phenetic approach to include 9 quantitative characters and to estimate the overall similarities between taxa. The phenetic analysis of morphological variations was rooted on midpoint (Figure 6B). As in the cladistic analysis, the phenetic clustering resulted in a good estimation of the phylogenetic relationships at the order rank with the monophyly of Phragmophora (approximately unbiased test value = 75) and Aphragmophora (78) and for assigning a species to the correct family. The phenetic approach was more congruent to molecular trees than the cladistics. It yielded the sister-group relationships between *X. sorbei* and Eukrohniidae (75), congruent relationships within Spadellidae with basal *P. gotoi*, *Spadella* monophyletic and sister group relationships between *S. ledoyeri* and *S. valsalinae. P. draco* was also found nested in a group comprising *S. bipunctata* and two *Parasagitta* species (87). Apart from the case of Heterokrohniidae and Krohnittidae, each having a single representative in the analysis, the remaining families, Spadellidae and Eukrohniidae, were found to be natural groups with high support (98 and 82 respectively). Sagittidae were not recovered not only because of the inclusion of *P. draco* but also that of *Krohnitta subtilis* as sister to *Serratosagitta* species. However, the low support for this grouping (63) raises questions over this mongrel assemblage. All Sagittidae genera except *Parasagitta* were monophyletic and most nodes picturing their relationships received good support. Similar to the molecular and morphological cladistic analyses, the morphological phenogram yielded a close relationship between *Ferosagitta* and *Aidanosagitta*. However, the three main Sagittidae lineages highlighted in the molecular trees were not recovered.

### Morphometrics

A phenogram was constructed on the basis of body shape similarities, using the Riemannian shape distance ρ, computed on all species pairs (see Additional file 3 for comparison between different families). The purpose was not to give more insights about chaetognaths relationships but rather to test whether the two pairs of lateral fins observed in Sagittidae come from the division of a single fin or from the neoformation of the anterior pair. The phenogram of body shape similarities was more congruent with the molecular data when it was constructed using the anterior end of the posterior lateral fin as homologous to the anterior end of the unique lateral fin of species having only one pair (PH1; agglomerative coefficient = 0.84; p = 0.0098***, Additional file 4A) than when it had been constructed with the anterior end of the anterior lateral fin as homologous to the anterior end of the unique lateral fin of species having only one pair (PH2; agglomerative coefficient = 0.89; p = 0.4537, Additional file 4B).

## Discussion

### A reassessment of chaetognaths relationships

The species studied in the present report belong to the Aphragmophora (Sagittidae, Krohnittidae and Pterosagittidae) and Phragmophora which have been divided in Biphragmophora (Heterokrohniidae) and Monophragmophora (Spadellidae and Eukrohniidae) [24]. We were therefore able to discuss the evolutionary history of six of the nine traditional families of the phylum (according to the views of Casanova [24] and Bieri [16]). The monophyly of the Krohnittidae cannot be debated because only one of the three known species, *Krohnitta subtilis*, is included in the present analysis. The same applies to Heterokrohniidae represented here only by *Xenokrohnia sorbei*. The Eukrohniidae and Spadellidae families have been confirmed, but not the Sagittidae and Pterosagittidae (a family comprising only one species, *Pterosagitta draco*). Indeed, the Sagittidae *sensu stricto* is a paraphyletic assemblage from which *P. draco* derives.

We observed poorly resolved basal nodes in the LSU rRNA trees and to a lesser extent in the SSU rRNA trees as well as a lack of relationships accuracy within Sagittidae. However, when molecular analyses were based on the concatenation of the two paralogous SSU rRNA class I and class II and LSU rRNA class I the resolution for deep branching nodes and the accuracy of relationships within Sagittidae were improved (see Figure 5). This indicates that when the amount of molecular data increases the phylogenetic signal does too. Thus, the low resolution of the single rRNA gene tree reconstructions is not due to a recent acceleration of diversification within Chaetognatha, as previously proposed [18,27], but rather to the short length of the aligned DNA segments.

Morphology and molecules produced some mixed phylogenetic results. This incongruence can be resolved by allowing several convergent losses and/or reversions of morphological characters. The lack of a character cannot be rigorously coded in cladistic due to the lack of primary homology hypothesis especially in a clade with few fossils and inappropriate outgroup. One consequence is that the amount of convergent losses can be underestimated. In such a situation, a phenetic approach should give a better estimation of the evolutionary history. In Chaetognatha, quantitative and qualitative morphological variations based on the degree of overall similarities have been more congruent with molecular topologies than cladistic did, especially concerning the relationships within Spadellidae and the position of *X. sorbei* as sister to Eukrohniidae. The position of *K. subtilis*, the only Krohnittidae relative, is also unstable according to the method used: either at the basis of all Sagittidae (cladistics on morphological data and molecular data) or sister group to *Pseudosagitta* within Sagittidae (phenetics on morphological data). Such an unstable position could be explained by an independent evolution of Krohnittidae during a long period from an early stage of Aphragmophora [21]. Finally, most inconsistencies observed between molecular and morphological approaches concern the Sagittidae relationships, which shows that there is no linear relationship between the degree of morphological divergence and the time of divergence within this family.

### Phragmophora – Aphragmophora split and the Ctenodontina/Flabellodontina hypothesis

The clade Sagittidae + *P. draco* as sister group to Krohnittidae is highly supported by our molecular and morphological cladistic analyses and revives the Aphragmophora, a clade invalidated by Papillon et al*.* [18]. However the conclusion these authors made was mainly based on the positioning of *P. draco* within Spadellidae, which was in agreement with morphometry and body appearance [25]. However, the present molecular results demonstrate that the previous *P. draco* sequence was likely to be a contaminant. Moreover, our morphological analyses do not support the Dallot and Ibanez [25] conclusion, highlighting the convergence of some morphological traits between Spadellidae and *P. draco*. It is interesting to note that on the basis of posterior lateral fins restricted to the tail observed in *Demisagitta demipenna* (firstly described as *Aidanosagitta demipenna* [31]), Bieri [17] pointed out a possible relationships between *P. draco* and some species belonging to Sagittidae. He noted that if *D. demipenna* were to lose the anterior fin and develop a pair of large floating bristles, the species would be included into the *Pterosagitta* genus. However, according to the first description [31] and a subsequent revision [21], even the position of the posterior lateral fins of *D. demipenna* is unique in Sagittidae and similar to the one-fin species *P. draco*, other characters conform to those of *Aidanosagitta*. Finally, and more importantly, the inclusion of *P. draco* within Sagittidae is corroborated by a recent report on the organisation of the chaetognath nervous system [32]. Indeed, this study showed that the RFamidergic pattern of *P. draco* is similar to that of several Sagittidae species when compared to several Spadellidae species.

Traditionally, authors who proposed internal systematics in Chaetognatha [16,17,21,22,24-27] identified two major groups; mainly on the basis of the presence or absence of transverse muscles - the phragms. Throughout this debate on chaetognath evolutionary trends, most authors agreed to consider the presence of phragms as a plesiomorphic state [21,24] but with slightly different hypotheses. Spadellidae were believed to have given rise to the Eukrohniidae and Heterokrohniidae according to Tokioka [21] whereas Casanova [24] considered the Heterokrohniidae as the chaetognaths that retain the highest number of plesiomorphic character states. Only Salvini-Plawen [26] suggested a radically different scenario which contradicted the primitiveness of phragms and identified Pterosagittidae as the sister group to all remaining families. Our results favour the ancestrality of Phragmophora and do not support Salvini-Plawen’s hypothesis since *P. draco* appears to be a highly specialised and homoplasic member of Sagittidae - as shown by various features such as the loss of the anterior lateral fins, the position of posterior lateral fins, the type of corona ciliata and a high trunk/tail length ratio. So far, the exact functional significance of phragms is unknown but their presence is correlated with a benthic lifestyle [21,22,26,33]. Indeed, the creeping and predatory activity on the sea bed requires more complicated movement than the pelagic niche does. One exception concerns the pelagic Eukrohniidae which exhibit phragms in the trunk, however these are vestigial structures believed to be functionless [22].

According to the rooted topology obtained on the basis of both paralogy classes of SSU rRNA genes (see Additional file 1), our analyses uphold the monophyly of the Aphragmophora but contradict that of the Phragmophora. The Phragmophora appear paraphyletic - a typical situation when a clade is defined on the basis of a plesiomorphic character state. However, the morphological cladistic approach shows that the Aphragmophora are only defined by the lack of phragm or the scarce development of glandular structures on the body surface, which leaves us in an unsatisfactory situation. Based on the current knowledge of gross morphology, histology, cytology and neuroarchitecture of chaetognaths, it is simply impossible to describe any noteworthy apomorphic feature of Aphragmophora. Finally, the Aphragmophora have been divided into two suborders [21,22]: Flabellodontina (Krohnittidae) and Ctenodontina (Sagittidae + Pterosagittidae). Our results show that this supplementary subdivision is unnecessary and undermines the hypothesis based on the structure of the cephalic armature that Ctenodontina could be closer to Phragmophora than to Krohnittidae [22].

### Validity of Biphragmophora and Monophragmophora

**Figure 7:**
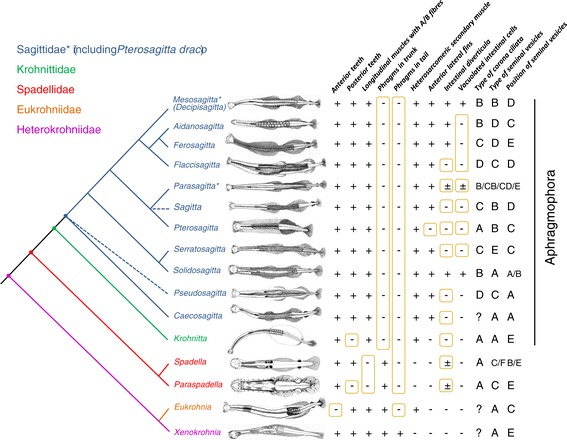
**Overview of the current phylogenetic relationships of Chaetognatha as reconstructed on the topology resulting from concatenated nuclear ribosomal genes (see Figure** **5** **).** Asterisks indicate clades that have been modified from the results obtained in the present study. The dotted lines characterize an unresolved branching. We propose to revive the name *Decipisagitta* instead of *Mesosagitta* (*i.e.*, *Decipisagitta decipiens, D. sibogae and D. neodecipiens)*, a genus proposed by Bieri [17]. The fourth and last species of *Mesosagitta, M. minima*, being now included in *Parasagitta*, the genus *Mesosagitta* does not exist anymore. On the right, the surrounded characters states indicate putative loss events. Illustrations after: Alvariño [37,38]; Casanova [70-72]; Winkelmann et al. [74].

The morphological analysis supports the division of Chaetognatha into three monophyletic groups, the Biphragmophora, Monophragmophora and Aphragmophora [24]. However, although the monophyly of the Biphragmophora (Heterokrohniidae) cannot be assessed because only one species has been included in our analyses, none of our molecular or morphological trees show that Monophragmophora (Spadellidae and Eukrohniidae) could be a natural group. All the tree topologies obtained suggest that the lack of phragms in the tail is a homoplasic state, which is observed independently twice in Eukrohniidae and Spadellidae. Yet, the grouping of Eukrohniidae and Heterokrohniidae is well supported for all molecular datasets and morphological phenetic analyses. These results are in accordance with a recent barcoding analysis showing a close relationship between *Heterokrohnia* and *Eukrohnia* species in respect to Sagittidae [28]. However, no morphological synapomorphy that could define the molecular clade constituted by *X. sorbei* + Eukrohniidae was identified. Indeed, the morphological character states shared by the representatives of these two families are plesiomorphic or homoplasic (a high trunk/tail length ratio also observed in Spadellidae and *P. draco*). Moreover, the Eukrohniidae family is traditionally only defined by the lack of anterior teeth (Figure 7). Thus, to definitely conclude such a sister-group relationship between these two families, a broader taxonomic sampling is needed. Deep benthoplanktonic representatives, such as the putative ancestral Heterokrohniidae, meso-bathyplanktonic Eukrohniidae and representative of *Hemispadella* genus, a link between the families Heterokrohniidae and Spadellidae [34], need to be studied for a better taxonomic coverage of Phragmophora.

### Bieri’s nomenclature and Sagittidae relationships

Despite the complexity of the distribution of some morphological characters, which poses problems when assessing the relationships within Sagittidae, most of the new genera proposed by Bieri [16] were supported by the molecular trees. Our results unambiguously confirm the monophyly of *Serratosagitta*, *Pseudosagitta*, *Flaccisagitta* and *Ferosagitta,* and to a lesser extent the validity of large and heterogeneous assemblages such as *Aidanosagitta* and *Parasagitta*. The relationships between species belonging to *Parasagitta* cannot be resolved on the basis of morphological analyses and received low support in the molecular trees. The morphology of this genus remains one of the most heterogeneous on the basis of several diagnosis characters which are prone to homoplasy (Figure 7): the structure and position of seminal vesicles, the presence/absence of intestinal diverticula, the presence/absence of intestinal vacuolated cells, the presence/absence of rayless zone in lateral fins and the structure of the corona ciliata if considering the inclusion of *Mesosagitta minima*. The status of *Solidosagitta* is still pending because only one species has been studied using SSU rRNA paralogous genes. However, our analyses based on morphological data and LSU rRNA sequences include two species and favour the validity of this latter genus.

Our molecular results divide the Sagittidae family into three major lineages (Figure 7): *Serratosagitta* + *Solidosagitta*, *Sagitta* + *Pterosagitta* + *Parasagitta* (including *M. minima*) and *Flaccisagitta* + *Aidanosagitta* + *Ferosagitta* + *Mesosagitta decipiens*. Species with vacuolated intestine (character #10) are distributed in these three lineages. This supports the opinion of Dallot [35] who considered the vacuolated species plesiomorphic on the basis of their general morphology and the structure of their seminal vesicles. This also strongly suggests that the ability to develop large intestinal vacuolated cells has been lost in numerous extant Sagittidae species. The grouping of *M. decipiens*, *Aidanosagitta*, *Ferosagitta* and *Flaccisagitta* receives high support in molecular phylogenies. Morphological analyses only support close relationships between *Ferosagitta* and *Aidanosagitta*. Moreover, some morphological characters are congruent with the association between *Aidanosagitta* and *M. decipiens*: the corona ciliata begins below eye level (type B [22]) and intestinal diverticula are present in *Mesosagitta* (*Decipisagitta*), *Aidanosagitta* and *Ferosagitta*. However, these characters isolate *Flaccisagitta* from the rest of the group: the corona ciliata is short and confined to the head, starting just behind the brain and stretching to the neck (type D [22]) and intestinal diverticula are absent. Kinship between *Sagitta bipunctata* and *Parasagitta* species are highly supported by rRNA data and has been previously proposed by several authors. According to Tokioka, [22], these chaetognaths display a similar extended corona ciliata (type C). Moreover, Furnestin [36] and Dallot [35] also suggested such affinities on the basis of the structure and position of lateral fins and number of teeth and hooks. An important incongruence between molecular and morphological analyses is the sister-group relationships between *Flaccisagitta* and *Pseudosagitta*, a result yielded by morphology but invalidated by all molecular trees. This group has previously been proposed by Tokioka but not all authors agree to bring these species in a same clade. Several authors [16,36-38] suggested that *lyra-gazellae-maxima* was undeniably a coherent group gathered in *Pseudosagitta* while *Flaccisagitta hexaptera* constituted the sister species to *F. enflata*. Finally, the morphological similarities between *Flaccisagitta* and *Pseudosagitta* could be linked either to a specialised form highly adapted to the oceanic plankton (thin primary muscles, flaccid body, not wholly rayed lateral fins with gelatinous masses) or should be considered as plesiomorphic states among Sagittidae (corona ciliata type D and seminal vesicles type C).

When taken together, these remarks emphasize the need to better define the morphological and anatomical boundaries between the traditional genera of Sagittidae by re-evaluating ancestral states and homologies of important traditional diagnostic characters at the histo- and cytological levels (for instance the nervous and muscular systems, the corona ciliata, the seminal vesicles and the fins).

### Taxonomic notes on the genera Parasagitta/Occulosagitta and Mesosagitta/Decipisagitta

In his attempt to improve the Sagittidae systematic, Bieri [17] also noticed the heterogeneity of several Sagittidae genera and modified his own classification by creating six new genera. Two species included in our analyses are concerned by these modifications: *Parasagitta megalophthalma* and *Mesosagitta decipiens* which were respectively renamed by Bieri as *Occulosagitta megalophthalma* and *Decipisagitta decipiens*. First, *Mesosagitta* as a natural group is contradicted. Indeed, in our molecular analyses, *Mesosagitta minima* always branches without any ambiguity within the *Parasagitta* genus while *M. decipiens* exhibits close relationships with *Flaccisagitta*, *Aidanosagitta* and *Ferosagitta*. Second *P. megalophthalma* always shows a close relationship with *Parasagitta elegans* whatever the molecular tree considered. Thus we propose (*i*) to invalidate the *Occulosagitta* genus, (*ii*) to rename *Mesosagitta minima* as *Parasagitta minima* and (*iii*) to gather the remaining *Mesosagitta* species (*M. decipiens*, *M. neodecipiens*, *M. sibogae*) into the new genus *Decipisagitta*.

### Phylogenetic consensus from molecules and implication for chaetognaths evolutionary trends

Paleontological evidences have demonstrated the existence of chaetognaths not only in the middle Cambrian Burgess Shale biota [39] but also in the earlier Cambrian Chengjiang biota [3] with morphological features almost identical to extant species. The discovery of new deep species [24] leads to the conclusion that Heterokrohniidae (Biphragmophora) is the family that presents the highest number of plesiomorphic characters (*i.e.,* the most primitive group *sensu* Casanova). However, our analysis raises the question of whether the lack of phragms in the tail of Spadellidae and Eukrohniidae is derived and due to convergence or represents a homologous and plesiomorphic character. The answer has far reaching consequences for our understanding of evolutionary pathways in chaetognaths. For instance, since phragms are considered important for creeping forms but are unnecessary for species that proceed by movements in the water column, this question is related to whether the stem chaetognaths were hyperbenthic or holoplanktonic species. The molecular and morphological phylogenetic results we obtained rather suggest that the Eukrohniidae and Spadellidae (Monophragmophora in Casanova’s hypothesis) exhibit the most primitive state, a scenario that needs only two evolutionary steps (one acquisition of phragms in the tail of Heterokrohniidae and one loss in the trunk of Sagittidae) against three steps when considering two parallel losses in the tail of Eukrohniidae and Spadellidae respectively, followed by one loss in the trunk of Sagittidae. However, we shall see that several arguments are in favour of the latter scenario. First, morphology and body ratios of specimens found in Chengjiang biota suggest that the chaetognaths from lower Cambrian were planktonic with ecological preferences for hyperbenthic niches close to the sea bottom [3]. Among extant genera, those showing the closest ecological features are the hyperbenthic Heterokrohniidae. Interestingly, their ecology is still observed in one species of Eukrohniidae, *Eukrohnia calliops* [16,40]. Second, phragms have been identified in trunk and tail of specimens from Cambrian Burgess Shale biota [39]. Finally, the stable environment of deep oceanic waters has likely delayed the body plan evolution and could explain the conservation of some ancestral morphology in the extant deep benthoplanktonic Heterokrohniidae. All summed up, it is reasonable to assume that an arrangement of transverse muscles in trunk and tail should be regarded as the most primitive state in chaetognaths. In hypothesizing such a complex Heterokrohniidae-like ancestor, one must postulate the loss of many structures and a body plan simplification during the evolutionary history of Chaetognatha (Figure 7).

In our scenario, an important split yielded two clades with different ecological niches, the strictly benthic Spadellidae and the holoplanktonic Aphragmophora lineages (Figure 7). This hypothesis contradicts the ancestrality of Spadellidae [22]. According to the comparative studies of the muscles in Chaetognatha, Spadellidae are highly derived and underwent important modifications of their muscular apparatus [33]. The structure of their primary muscles which lack B fibres is characteristic of benthic species and is derived from AB fibres typology. Combined with our results, there is therefore strong evidence that the Spadellidae ancestor was planktonic, partly linked to the sea bed, and adapted secondarily to a strict benthic lifestyle. The highly specialized status of this family is shown by its high number of synapomorphies (characters #2, #5, #6 and #8; Additional file 2).

The Aphragmophora diversification does not display any morphological novelty and highlights a new case of body plan simplification with the loss of phragms in the trunk (Figure 7). The last Aphragmophora ancestor divided into two lineages, giving rise to the current Krohnittidae and Sagittidae families. The Krohnittidae family retained some ancestral traits such as one pair of lateral fins on the trunk and tail, with the anterior end and posterior rear end at equal distance from the caudal septum (character #2) but also developed numerous autapomorphic features. They exhibit abruptly curved hooks (character #11) and anterior teeth arranged in a fan shape (character #22) and they lack posterior teeth (character #20). The peculiarity found in teeth and hooks of *Krohnitta* species reveals a high level of specialisation [21] and points to an independent evolution of Krohnittidae that started during the early stages of Aphragmophora cladogenesis as demonstrated by our results. Interestingly, the arrangement and shape of the anterior teeth of *Sagitta nairi*, a Sagittidae recently described [41] are similar to those of the genus *Krohnitta* suggesting a possible convergence between these unrelated species. The second Aphragmophora family, the Sagittidae, is defined by two pairs of lateral fins. *Caecosagitta* and *Pseudosagitta* can be recognized as two early off-shoots of Sagittidae. Dallot and Ibanez [25] have already proposed the isolation of *Pseudosagitta lyra* and suggested the possibility that its membership to the Sagittidae is dubious. Our results do not support such exclusion.

An important question considering body plan variation in Chaetognatha is concerned with fin evolution. More precisely, did the anterior and posterior lateral fins of Sagittidae originate from the division of the single large fin observed in Heterokrohniidae and Eukrohniidae? Casanova and Moreau [42] noted some similarities between the posterior lateral fin of species belonging to *Pseudosagitta* genus and the unique lateral fin of those belonging to Eukrohniidae, the posterior extremities of which are only slightly apart from the bodywall. The posterior and anterior lateral fins of *Pseudosagitta* are connected by a tegumentary bridge which reinforces the idea that the two lateral fins would have formed after the incomplete division of a unique large one. However, our morphometric analysis shows that the anterior lateral fin of two-fin species does not result from the division of a unique lateral fin but from a neo-formation after a backward movement of the anterior end of the unique fin. In other words, the posterior lateral fin of Sagittidae is homologous to the unique lateral fin of the other families. This evolutionary step constitutes a rare case of increase in body plan complexity at the anatomical level in Chaetognatha. While the possession of two pairs of lateral fins was recognized as a good synapomorphy for Sagittidae after Tokioka’s classification, the inclusion of *P. draco* which exhibits only one posterior pair raises doubts about its validity. This means that the loss of the anterior fin did not constitute an evolutionary dead end for Sagittidae making the presence of one pair of fin a homoplasy.

As previously mentioned [18,33], the distribution of some morphological characters in Chaetognatha cannot be related to the phylogeny nor to the ecology suggesting a differential evolution of separate chaetognath organs (mosaic evolution). The prevalence of mosaic evolution can be demonstrated through the examination of character associations in extant chaetognaths (Figure 7). Different traits and outcomes are favoured by natural selection in different species and these evolutionary pathways might be responsible for the non congruence between some of the cladistic and phenetic analyses because when mosaic selection occurs the primitive or derived nature of character states cannot be deduced on the basis of their correlation with other character states which are believed to be primitive or derived [43]. Moreover, a common trend correlated with mosaic evolution is the prominence of homoplasy powered by common selective pressures as shown in many plants [44] and animals [45-47].

Such a combination of mosaic evolution and lack of fossil records can lead to persistent problems in interpreting relationships through morphological cladistic analysis [48].

### Ecologically-induced convergence in holoplanktonic chaetognaths

It is likely that all Aphragmophora and Eukrohniidae lineages are morphologically highly similar because of their holoplanktonic lifestyle in the pelagos [22,26] (Figure 7). For instance, in spite of their separate evolutionary history, Sagittidae, Krohnittidae and Eukrohniidae share features such as the common tendency towards a body with large surface to volume ratio, the trunk elongation, the reduction of epidermal glandular structures and the reduction/loss of phragms. Because these clades do not constitute a natural grouping, it is obviously a case of convergent evolution. Functional similarity is also present at the cytological level since all these holoplanktonic species exhibit the highest proportion of B fibres in their primary muscles and a heterosarcomeric organisation of their secondary muscles [33]. Among Sagittidae, the morphology of *P. draco* is more puzzling. There are numerous homoplasic reversions in this species since it exhibits a high trunk/tail length ratio and one small pair of lateral fins being restricted to the tail, a typical set of Spadellidae morphological features. This epipelagic species compensates the decrease of its surface/volume body ratio by developing a foamy epidermal collarette around most of the body combined with floating bristles.

Buoyancy ability represents an interesting area of investigation in holoplanktonic chaetognaths [49-52]. To decrease their specific gravity, Eukrohniidae show a hyper development of the unique pair of lateral fins and vestigial phragms, which are likely to be not functional and are confined to the most anterior part of the trunk [22]. A similar trend is observed in Krohnittidae with the development of large lateral fins and phragms that have totally disappeared. The Sagittidae adapted in a different way by creating a new pair of lateral fins. This acquisition constitutes an important and unique event to adapt to holoplanktonic lifestyle in Chaetognatha and could contribute to explain the successful current biodiversity of Sagittidae. It has been recently shown that the diversification of the Euthecosomata, which are important holoplanktonic molluscs, occurred in the context of an important turn-over in the marine planktonic community due to severe environmental changes that started from the Late Palaeocene [53]. Moreover, these morphological innovations correlated with climatic changes and species turn-over were largely shaped by shell buoyancy adaptation. One could postulate that a broad and recent diversification of Sagittidae occurred in the same evolutionary background. However, the paucity of fossils for these soft bodied invertebrates did not allow an efficient calibration of the divergence times of Chaetognatha lineages.

## Conclusions

Molecular analyses have highlighted the homoplasy of several traditional characters and the influence of lifestyle on morphology, particularly for chaetognaths that adapted to a pelagic environment. We also propose that Chaetognatha evolved mostly through simplification of a pre-existing body plan, rather than through an increase in complexity. This constitutes a shift of paradigm in the traditional understanding of the group’s evolution and prompts a re-appraisal of previous hypotheses concerning the morphological characters’ polarity and history. For example, it is reasonable to think that the loss of phragms and teeth could have occurred independently in different branches during chaetognath evolution. If the anterior and posterior rows of teeth are considered as homologous structures, the loss of one of these rows may be described as an event of parallel evolution. Another important traditional diagnostic character, the trunk/tail length ratio, can also be considered subject to homoplasy by reversion due to Bauplan limits (*Pterosagitta draco* versus Spadellidae) and convergence caused by an evolution in similar ecosystems (Sagittidae/Krohnittidae *versus* Eukrohniidae). Even the main Sagittidae synapomorphy represented by two pairs of lateral fins is homoplasic by reversion. Because of these numerous losses and homoplasic events, traditional morpho-anatomical traits may prove unhelpful with deciphering chaetognath relationships and morphological evolution with any certainty. Such a conclusion stresses the need for more data from molecular markers as well as from histo- and cytoarchitecture of the muscular apparatus [33] and the neurosensorial system [32,54]. Because of the scarcity of novelties at the anatomical level, it becomes needed to explore their body plan variations from the tissue level to the cell level. Considering the pivotal phylogenetic position of Chaetognatha within bilaterians, it is of primary importance to reconstruct their ground pattern. Future studies need to focus on a set of new characters based on a broad range of taxa including specimens belonging to meso-bathypelagic and deep benthoplanktonic genera. The use of an expanded taxonomic dataset combined with appropriate observation (*i.e.*, with transmission electron microscopy, immunohistochemistry combined with confocal laser scanning microscopy and Next Generation Sequencing) will be crucial in improving the understanding of Chaetognatha’s diversification processes.

## Methods

### Collection, species diagnosis and taxonomic sampling

Specimens were collected from a broad geographical range sampled during the circum-global *Tara* Ocean expedition and also from other regional missions (Table 1). Table 2 shows the sequences used in the present study already available in public databases. Morphological identifications of specimens were performed using Wild M5 and Nikon SMZ 645 stereoscopic microscopes, and combined with ecological and molecular data from previous literature. For morphological data, comparisons with original descriptions were undertaken. When different species exhibited a very close gross morphology and similar number of teeth and hooks, only mature adults were chosen because one of the key features for the diagnosis of chaetognaths in ethanol is the shape and position of seminal vesicles disposed on each side of the tail. For ecological data, specimens’ habitats and depths were taken into account. For molecular data, acquired DNA sequences from the study were compared against previously obtained sequences in GenBank. Finally, if morphology for specimens did not fit perfectly with the described species, we named species using “cf.” accordingly. In this manuscript, we used the taxonomic nomenclature given by Bieri [16,17] who renamed several species and created nine genera of Sagittidae.

**Table 1:** **Details of the sequences and GenBank accession numbers obtained from this study**

**Species**	**Code**	**SSU rRNA I**	**SSU rRNA II**	**LSU rRNA I**	**LSU rRNA II**	**Mission/Collector**	**Origin**
***Krohnitta subtilis***	011	KM519840	KM519864			DIVA3 St ME 791/540.1	Med. E
***Krohnitta subtilis***	039	KM519842	KM519863	KM519928		TARA St 64	Ind. N
***Krohnitta subtilis***	058	KM519841	KM519862			TARA St 76	Atl. SW
***Krohnitta subtilis***	068			KM519927		TARA St 132	Pac.
***Pterosagitta draco***	026	KM519820	KM519898	KM519906		Florida/CRER 2 St 91	Atl. W
***Pterosagitta draco***	076	KM519818				Florida /CRER 2 St 42	Atl. W
***Pterosagitta draco***	001	KM519819				Tulear/ M. Pagano	Ind. N
***Pterosagitta draco***	002		KM519896	KM519905		Tulear/M. Pagano	Ind. N
***Pterosagitta draco***	003		KM519897			Florida/CRER 2 St 42	Atl. W
***Parasagitta elegans***	004	KM519817				Norway/F. Norrbin	Atl. N
***Parasagitta elegans***	005		KM519901			Norway/F. Norrbin	Atl. N
***Parasagitta elegans***	038	KM519815	KM519900			Norway/F. Norrbin	Atl. N
***Parasagitta elegans***	075	KM519816	KM519899	KM519923		Norway/F. Norrbin	Atl. N
***Parasagitta setosa***	012	KM519810				France/Y. Perez	Med. NW
***Parasagitta setosa***	028	KM519809				France/Y. Perez	Med. NW
***Parasagitta setosa***	045	KM519808		KM519920		France/Y. Perez	Med. NW
***Parasagitta friderici***	013	KM519811		KM519922		France/Y. Perez	Med. NW
***Parasagitta friderici***	046	KM519812		KM519921		France/ Y. Perez	Med. NW
***Serratosagitta pacifica***	025	KM519827	KM519872			TARA St 34	Red Sea
***Serratosagitta pacifica***	031	KM519826	KM519871	KM519904		TARA St 34	Red Sea
***Serratosagitta pacifica***	032	KM519825	KM519873			TARA St 65	Ind. N
***Serratosagitta serratodentata***	014	KM519830	KM519870			MSN 14/1 St 1159	Med.
***Serratosagitta serratodentata***	044	KM519828	KM519869			TARA St 64	Ind. N
***Serratosagitta serratodentata***	047	KM519829	KM519868			TARA St 16	Med. C
***Serratosagitta tasmanica***	006	KM519831				France/ Y. Perez	Atl. E
***Serratosagitta tasmanica***	043	KM519832	KM519866	KM519902		TARA St 66	Ind. N
***Serratosagitta tasmanica***	049		KM519865			TARA St 16	Med. C
***Serratosagitta tasmanica***	063	KM519833	KM519867	KM519903		TARA St 79	Atl. SW
***Pseudosagitta lyra***	007	KM519837				France/Y. Perez	Atl. E
***Pseudosagitta lyra***	008		KM519858			France/Y. Perez	Atl. E
***Pseudosagitta lyra***	029	KM519835	KM519857			TARA St 65	Ind. N
***Pseudosagitta lyra***	030	KM519836	KM519856			TARA St 66	Ind. N
***Pseudosagitta lyra***	023	KM519838	KM519854			TARA St 23	Med. C
***Pseudosagitta lyra***	024		KM519855			TARA St 15	Med. W
***Pseudosagitta gazellae***	055		KM519859			TARA St 86	Ant.
***Pseudosagitta gazellae***	056	KM519839	KM519860			TARA St 86	Ant.
***Flaccisagitta hexaptera***	018	KM519792	KM519880			MSN 14/1 St 1159	Med. E
***Flaccisagitta hexaptera***	035	KM519791	KM519881			TARA St 52	Ind. N
***Flaccisagitta hexaptera***	036	KM519790	KM519883			TARA St 64	Ind. N
***Flaccisagitta hexaptera***	048	KM519789	KM519882			Florida CRER2 St 42	Atl. W
***Flaccisagitta enflata***	021	KM519795	KM519878			TARA St 18	Med. W
***Flaccisagitta enflata***	022	KM519794	KM519877			TARA St 15	Med. W
***Flaccisagitta enflata***	033	KM519793	KM519876			TARA St 34	Red Sea
***Flaccisagitta enflata***	034	KM519796	KM519879			TARA St 64	Ind. N
***Aidanosagitta cf. oceania***	010	KM519801		KM519914		Tulear/M. Pagano	Ind. N
***Aidanosagitta cf. septata***	069	KM519802	KM519891	KM519917		TARA St 58	Ind. W
***Aidanosagitta regularis***	066	KM519803	KM519892	KM519915		TARA St 109	Pac. E
***Aidanosagitta regularis***	072	KM519804				TARA St 130	Pac.
***Aidanosagitta regularis***	073			KM519916		TARA St 130	Pac.
***Aidanosagitta neglecta***	042	KM519805	KM519894	KM519913		TARA St 50	Ind. C
***Aidanosagitta neglecta***	041	KM519806	KM519893	KM519912		TARA St 32	Med. E
***Mesosagitta decipiens***	067	KM519807	KM519884	KM519907		TARA St 32	Red Sea
***Mesosagitta minima***	038	KM519813	KM519885	KM519909		TARA St 66	Ind. N
***Mesosagitta minima***	037	KM519814	KM519886	KM519908		TARA St 64	Ind. N
***Solidosagitta marri***	053	KM519823	KM519874	KM519911		TARA St 86	Ant.
***Solidosagitta marri***	054	KM519824	KM519875			TARA St 85	Ant.
***Caecosagitta macrocephala***	052	KM519834	KM519861	KM519910		Florida/C. Guigand	Atl. N
***Ferosagitta ferox***	064	KM519800	KM519887	KM519925		TARA St 100	Pac. E
***Ferosagitta ferox***	071	KM519799	KM519889	KM519924		TARA St 125	Pac.
***Ferosagitta ferox***	074	KM519798	KM519888			TARA St 125	Pac.
***Ferosagitta cf. tokiokai***	065	KM519797	KM519890	KM519926		TARA St 100	Pac. E
***Sagitta bipunctata***	019	KM519821		KM519918		TARA St 17	Med. W
***Sagitta bipunctata***	020	KM519822	KM519895	KM519919		TARA St 15	Med. W
***Spadella valsalinae***	015	KM519848				Croatia/C. Müller	N. Adriatic
***Spadella valsalinae***	016	KM519846				Croatia/C. Müller	N. Adriatic
***Spadella valsalinae***	059	KM519847		KM519929		Croatia/C. Müller	N. Adriatic
***Spadella cephaloptera***	060	KM519844		KM519930		Ibiza/Y. Perez	Med. NW
***Spadella cephaloptera***	061	KM519845				France/Y. Perez	Med. NW
***Spadella cephaloptera***	062	KM519843				France/Y. Perez	Med. NW
***Xenokrohnia sorbei***	009	KM519849		KM519931		France/Y. Perez	Atl. E
***Eukrohnia bathypelagica***	051	KM519851				France/Y. Perez	Atl. E
***Eukrohnia bathypelagica***	057	KM519850			KM519933	TARA St 85	Ant.
***Eukrohnia hamata***	017	KM519853				Norway/F. Norrbin	Atl. N
***Eukrohnia hamata***	050	KM519852				Norway/F. Norrbin	Atl. N
***Eukrohnia fowleri***	070				KM519932	Florida/C. Guigand	Atl. N

**Table 2:** **Details of the sequences and GenBank accession numbers obtained from previous studies and used in the present analysis**

**Species**	**SSU rRNA I**	**SSU rRNA II**	**LSU rRNA I**	**LSU rRNA II**	**Collector**	**Origin**
***Parasagitta megalophthalma***	DQ351878	DQ351901			Y. Perez	Med. NW
***Parasagitta elegans***	Z19551				M. Telford	Pac. NW
***Parasagitta elegans***			Z77108		Q. Bone	Atl. N
***Parasagitta setosa***		DQ351900			J.P. Casanova	Atl. N
***Parasagitta setosa***			Z77120		J.P. Casanova	Atl. E
***Parasagitta setosa***			Z77121		V. Øresland	Atl. N
***Serratosagitta serratodentata***			Z77119		J.P. Casanova	Atl.
***Serratosagitta tasmanica***	DQ351893				F. Norrbin	Atl. N
***Pseudosagitta lyra***	DQ351880	DQ351892			Y. Perez	Med. N
***Pseudosagitta lyra***			Z77114		E. Thuesen	Pac. E
***Pseudosagitta maxima***			Z77118		F. Kurbjeweit	Ant.
***Pseudosagitta gazellae***			Z77112		F. Kurbjeweit	Ant.
***Flaccisagitta hexaptera***			Z77113		M. Terazaki	Pac. W
***Flaccisagitta enflata***	DQ351877				B. Thomassin	Ind. W
***Flaccisagitta enflata***			Z77109		J.P. Casanova	Atl.
***Flaccisagitta enflata***			ZZ110		M. Terazaki	Pac. W
***Aidanosagitta crassa***	D14363				T. Goto	Pac. W
***Aidanosagitta crassa***			Z77107		S. Nagasawa	Pac. W
***Aidanosagitta neglecta***	DQ351882				B. Thomassin	Ind. W
***Mesosagitta decipiens***	DQ351881	DQ351895			Y. Perez	Med. NW
***Solidosagitta zetesios***			Z77122		E. Thuesen	Pac. E
***Solidosagitta marri***			Z77117		F. Kurbjeweit	Ant.
***Caecosagitta macrocephala***			Z77115		E. Thuesen	Pac. E
***Caecosagitta macrocephala***			Z77116		M. Terazaki	Pac. W
***Xenokrohnia sorbei***	DQ351888	DQ351902			Y. Perez	Atl. E
***Ferosagitta ferox***			Z77111		M. Terazaki	Pac. W
***Ferosagitta robusta***				Z77130	M. Terazaki	Pac. W
***Sagitta bipunctata***	DQ351890	DQ351894			J.P. Casanova	Atl. E
***Sagitta bipunctata***				Z77127	J.P. Casanova	Atl.
***Paraspadella gotoi***	D14362				T. Goto	Pac. W
***Spadella ledoyeri***	DQ351883	DQ351899			C. Lejeusne	Med. N
***Spadella cephaloptera***	DQ3351884	DQ351897			Y. Perez	Med. NW
***Spadella cephaloptera***				Z77129	D. Dixon	Atl. NE
***Eukrohnia bathypelagica***	DQ351886	DQ351896			Y. Perez	Atl. E
***Eukrohnia hamata***	DQ351887				F. Norrbin	Atl. N
***Eukrohnia hamata***			Z77105		H. Kapp	Atl. E
***Eukrohnia hamata***			Z77106		F. Kurbjeweit	Ant.
***Eukrohnia fowleri***		DQ351889			Y. Perez	Atl. E
***Eukrohnia fowleri***			Z77103		H. Kapp	Atl. E
***Eukrohnia fowleri***				Z77123	E. Thuesen	Pac. E
***Eukrohnia fowleri***				Z77124	H. Kapp	Atl. E
***Eukrohnia fowleri***				Z77125	H. Kapp	Atl. E
***Eukrohnia fowleri***				Z77126	M. Terazaki	Pac. W

### DNA extractions, PCR amplifications and sequencing

All specimens were placed in 80% ethanol for preservation. The genomic DNA was then extracted using the DNAeasy kit (Qiagen, Valencia, CA) from pieces or entire individuals dried on filter paper and devoid of alimentary bolus to prevent contamination.

Because the whole ribosomal cluster in Chaetognatha is duplicated [18,27] two sets of specific primers for each paralogous SSU rRNA gene were used to amplify sequences of approximately 1800 bp (class I: 18SCI5′ TTGATGAAACTCTGGATAACTC and 18SCI3′ GGACCTCTCTACATCGTTCG) and 1200 bp (class II: 18SCII5′ TCGTCGGGGTCTCATCC and 18SCII3′ AGATACCTCGCAAAATCG). As we concentrated our efforts on the class I of LSU rRNA gene, which is the most represented class in public databases, only one set of primers previously described in [27] was used to amplify a fragment of approximately 500 pb: 28S5′ AAAGGATCCGATAGYSRACAAGTACCG and 28S3′ CCCAAGCTTGGTCCGTGTTTCAAGAC. Most of the sequences obtained with this couple of primers belonged to the class I but we also amplified LSU rRNA class II genes for several species.

PCRs were performed according to [27] in 50 μL volumes with the following reagents: 1× PCR buffer (Taq PCR core kit, Qiagen), 0.2 mM of each dNTPs mix, 0.5 mM of each primer, 2 to 4 μl (depending on DNA concentration) of extracted genomic DNA, and 1U of Taq polymerase. The PCR cycling parameters for amplification of LSU rRNA were: 95°C, 3 min, then 35 cycles of 95°C for 1 min, 50°C for 1 min, 72°C for 2 min.

For SSU rRNA, we used the following PCR program: 2 min at 92°C; 5 cycles of 92°C for 30 s, 48°C for 45 s, 48°C to 72°C for 80 s (ramp rate of 0.3°C/s) and 72°C for 90 s; 30 cycles of 92°C for 45 s, 48°C for 45 s, 72°C for 90 s; and a final extension time of 72°C for 7 min.

After amplification, all PCR fragments were purified with Wizard® SV Gel and PCR Clean-Up System (Promega, Madison, WI), cloned into pGemT-easy vector (Promega, Madison, WI) and sequenced in both directions using the T7 and SP6 primers with a ABI 96-capillary 3730XL sequencer at Eurofins genomics. 145 sequences have been deposited in GenBank under the following accession numbers:

- SSU rRNA class I: KM519789 - KM519853
- SSU rRNA class II: KM519854 - KM519901
- LSU rRNA class I: KM519902 - KM519931
- LSU rRNA class II: KM519932 - KM519933.

### Molecular phylogenetic analysis

Five data sets were used for molecular analyses: dataset 1 = LSU rRNA Class I and II, dataset 2 = SSU rRNA Class I and II, dataset 3 = SSU rRNA Class I, dataset 4 = SSU rRNA Class II and dataset 5 = SSU rRNA Class I, SSU rRNA II and LSU rRNA Class I concatenated sequences. The sequence alignments were established using CLUSTALX [55] and Muscle [56] implemented in Mega 5 and then further improved manually. The MODELTEST *v3.0b4* program [57] was used to identify the best model of DNA evolution for each of our dataset based on maximum likelihood (ML) and using Bayesian information criterion (BIC). We used Aliscore [58,59] to test the impact of highly heterogeneous sites that could negatively affect the phylogenetic reconstruction. We used the following parameters “-N” and “-N –r –w4” to remove heterogeneity sites. A Maximum Likelihood tree was estimated using the Nearest-Neighbour-interchange (NNI) option with Mega 5. A random starting tree was generated using the Neighbour-Joining method with the partial deletion option selected (75% site coverage cut-off). Topological robustness was investigated using 1000 non-parametric bootstrap replicates. Branches with bootstrap values higher than 70% were considered well supported [60]. We also performed Bayesian phylogenetic analyses using MrBayes 3.0b4 [61]. Each analysis consisted of 2.10^7^ generations with a random starting tree, default priors, the same set of branch lengths for each partition, and four Markov chains (with default heating values) sampled every 1000 generations. Adequate burn-in was determined by examining a plot of the likelihood scores of the heated chains for convergence on stationarity as well as the effective sample size (ESS) of values in Tracer 1.5 [62].

To test the impact of potential “noisy sites” we computed maximum likelihood phylogenetic analyses using PHYML aBayes 3.0.1 beta programme [63,64] on the LSU rRNA data set. We calculated two non-parametric branch supports (Bootstrap and SH-aLRT) and two parametric branch supports (aBayes and approximative likelihood ratio test, aLRT) as developed in [64,65]. We used bootstrap (bv) and aLRT (aLRTv) values and posterior probabilities (pp) to establish a criterion of “quality”. If bv was “low” but the other two were “high” then we considered a potential false negative support; if bv was “high” but the other two were “low” then we considered a potential false positive support.

We sometimes included sequences that were highly divergent, for instance from *Aidanosagitta crassa*, *Eukrohnia fowleri*, *Serratosagitta tasmanica* and, less frequently, from *Mesosagitta decipiens* because our primary goal was to accommodate the widest taxonomic and molecular dataset possible. This will provide a good foundation for future studies on chaetognaths evolution, but it may negatively impact our phylogenetic reconstruction. We removed some of these sequences in the concatenated dataset 5 to test whether they could produce artefacts in phylogenetic reconstructions.

### Morphological analysis

The data set considered here is constituted of the 34 species used in the molecular analysis. The following 32 characters were chosen on the basis of their traditional importance as key characters and their use in species diagnosis. These variables are a mixture of different types (see the full list below): 23 qualitative (binary and polytomic) and 9 quantitative (*e.g.*, lengths). These data are coming from a compilation of original descriptions and reviews [21,22,37,38,66-74]. Characters coding is presented in Additional file 2.

Qualitative characters (n = 23)

- C1- Body type: Flaccid =1; Rigid =2
- C2- Number and type of lateral fins: one long pair of lateral fins extended on the tail as well as on the most part of the trunk =1; one short pair on the trunk and tail, the anterior end at the level of the caudal septum =2; one short pair on the trunk and tail, with the anterior and the posterior ends at equal distance from the caudal septum =3; Two pairs of lateral fins =4
- C3- Tegumentary bridge connecting the anterior and posterior lateral fins: absent = 1; present =2
- C4- Phragms (transverse muscles): absent = 1; present in the trunk only =2; present in the trunk and tail =3
- C5- Type of phragms: supercontraction =1; normal contraction =2
- C6- Type of longitudinal muscles: only B fibre =1; A and B fibres =2
- C7- Type of secondary muscles: heterosarcomeric secondary muscles (He S) =1; homosarcomeric secondary muscles (Ho S) =2
- C8- Organisation of RFamide-like neurons: Type A (absence of D6 and X posterior neurons, absence of caudal loop) =1; Type B (presence of D6 and X posterior neurons with caudal loop) =2
- C9- Intestinal diverticula: absent = 1; present =2
- C10- Vacuolated intestinal cells: absent = 1; present =2
- C11- Type of hooks: gently curved =1; gently curved and serrated =2; abruptly curved =3
- C12- Type of seminal vesicles: elongated with a lateral opening =1; elongated and an anterior protruding part usually roundish =2; roundish or slightly oval with a lateral opening =3; elongated with an anterior opening =4; presence of small indentations =5; oval with bulb-like shape =6
- C13- Position of seminal vesicles (in respect to lateral and tail fins): touching neither lateral fins nor tail fin but closer to lateral fins =1; touching neither lateral fins nor tail fin but closer to tail fin =2; touching, or close to, lateral fins and well separated from tail fin =3; touching, or close to, tail fin and well separated from lateral fins =4; touching both lateral fins and tail fin =5
- C14- Ocular type: inverted =1; everted =2
- C15- Pigmented cell in the eye: absent = 1; present =2
- C16- Secretory ventral gland: absent = 1; present =2
- C17- Gelatinous masses in the lateral fins: absent = 1; present =2
- C18- Adhesive papillae: absent = 1; present on the ventral side of the body and fins =2; concentrated on adhesive appendages =3
- C19- Type of corona ciliata: Type A =1; Type B =2; Type C =3; Type D =4 (A–D, type of corona ciliata after Fowler [67] and Tokioka [21,22])
- C20- Number of teeth rows: one anterior row =1; one posterior row =2; two rows =3;
- C21- Epidermal glandular structures: Glandular structure on the body surface scarcely developed =1; numerous glandular structures on the body surface =2
- C22- Type of teeth: stout teeth arranged in fan shape =1; slender teeth arranged in comb-shaped =2
- C23- Ray less zone in the lateral fins: absent = 1; present =2

Quantitative characters (n = 9)

- C24- Trunk/tail length ratio (minimum value)
- C25- Trunk/tail length ratio (maximum value)
- C26- Position of the ventral nerve centre (in respect to the trunk length)
- C27- Minimum number of anterior teeth
- C28- Maximum number of anterior teeth
- C29- Minimum number of posterior teeth
- C30- Maximum number of posterior teeth
- C31- Minimum number of hooks
- C32- Maximum number of hooks

Qualitative morphological data were analysed using Paup* 4.0b10 under maximum parsimony (MP) with a heuristic search with 10 random taxon addition replicates followed by tree bisection and reconnection (TBR) branch swapping. All characters were treated as unordered and unweighted. ACCTRAN (accelerated transformation) and DELTRAN (delayed transformation) character optimization were both used to map the character changes and resolve ambiguous nodes. The g1 statistic was obtained using 1 000 000 random trees. Clade frequencies were obtained by 50% majority-rule consensus trees.

As many chaetognath lineages have been defined by the lack of a given structure, we also conducted a phenetic approach to integrate quantitative data and to estimate the degree of overall similarity information available (*i.e.,* the absence of a character as valuable phylogenetic information). In order to include all variables in a common analysis, we chose to treat the data set as quantitative by replacing each qualitative variable by its disjunctive table. Such a table contains as many columns as modalities: each column defining a binary variable of 1 if the modality is observed and 0 otherwise. After this operation we ended up with a table of 34 lines (taxa) and 59 columns (original variables for quantitative characters or binary score corresponding to a modality for qualitative ones). We considered the Euclidian distance between two taxa after scaling each column to one and performed a non-supervised hierarchical clustering using the Ward algorithm. As the number of columns is slightly higher than the number of taxa it is especially important here to access the uncertainty of the relationships obtained after the classification procedure. We did that using the re-sampling procedure implemented in the pvclust package [75] of R version 3.0.1 [76]. At each bifurcation of the classification, the variables significantly different between the two classes were identified. This was made by performing t-test for quantitative variables and chi-square test for qualitative variables that allowed us to characterize classes of the topology. Although the bootstrap probability test is very useful for tree selection, it is biased. The selection bias comes from comparing many trees at the same time and often leads to overconfidence in the wrong trees. So, we chose the approximately unbiased (au) test for assessing the confidence of tree selection - a method less biased than other methods such as bootstrap probability test [77].

### Geometric morphometrics

We carried out a geometric morphometric approach [78] to explore body shape variations among the species used in the molecular analysis. The aim of this method was to test two different primary homology hypotheses on the evolution of structure and number of lateral fins and to correlate body shape patterns in relation to different locomotor and environmental behaviours (*i.e.*, benthic versus pelagic) in Chaetognatha. Over the last three decades, systematic studies have often been complemented by geometric morphometrics, allowing the computation and visualization of global shape changes in organs or organisms. Procrustes superimposition is the most effective method for creating spatial graphical representations of shape variations [79].

The variation of the body shape patterns was statistically studied from morphotypes belonging to the six traditional clades identified in the molecular analysis: Heterokrohniidae, Eukrohniidae, Spadellidae, Pterosagittidae, Krohnittidae and Sagittidae. Illustrations of representative species used in this pilot study mostly come from the publications of Alvariño [37,38] who provided the most accurate drawings of chaetognaths with respect to their body shape proportions and the position of their ventral nerve centre, lateral fins and seminal vesicles. Other sources were Tokioka’s illustrations of *Aidanosagitta crassa* [69], Casanova’s illustrations of *Paraspadella gotoi* [71] and *Xenokrohnia sorbei* [72], the description by Dallot and Ducret [73] of *Parasagitta megalophthalma* as well as pictures of specimens belonging to the *Spadella* genus (*Spadella ledoyeri*, *Spadella cephaloptera* and *Spadella valsalinae*) by the authors of the present study.

Digital images were obtained with a flat bed scanner. Then, 20 landmarks were digitalized using TPSdig2 [80] (Additional file 5). When the depicted specimens were not straight, we used the following procedure to get landmark coordinates of straightened specimens: we first calculated the mid points between homologous points on the right and left side of the specimens. This series of points was then aligned on the x-axis, and the relevant landmark points repositioned with a Y coordinate of half the distance between the left and right points (Additional file 5). Only one side of the individual specimens was then used for further analyses. The shape variation was analyzed by the generalized Procrustes method using the R shape package [81]. Two primary homology hypotheses (PH) were tested: the anterior end of the posterior lateral fin in two fin species is homologous to the anterior end of the unique lateral fin in one-fin species (PH1), or the anterior end of the anterior lateral fin in two fin species is homologous to the anterior end of the unique lateral fin in one fin species (PH2). The full Procrustes distances between the conformations of each possible pair of species were computed in the two homology hypotheses. Dendrograms of landmark conformation similarities were computed by UPGMA. These hypotheses were tested to establish which of the two dendrograms from the morphometric data shows the higher agglomerative coefficient, and whether they were congruent with molecular data.

## Additional files

Additional file 1:
**Phylogeny of chaetognaths inferred from the SSU rRNA dataset 2 including both paralogy classes (138 sequences from 33 species: 80 class I and 58 class II sequences; 1679 base pairs long).** Support values obtained using different reconstruction approaches are indicated at nodes in the following order: maximum Likelihood bootstrap probabilities (bv), approximative likelihood ratio test (aLRTv) and Bayesian posterior probabilities (pp). Support values are displayed when bv/aLRTv ≥75 or pp ≥0.85. Node absence in a given method is indicated by -. Additional file 2:
**Morphological data matrix.** Unknown character states are indicated by NA and non-homologous characters are indicated by -. Additional file 3:
**Diagrammatic representation of morphometric measures used for the analysis of body shape similarities.** A: Superimposition of all the studied chaetognaths, altering the scales, to superimpose their anterior and posterior ends (points 1 and 20). B-F: same graphs family per family (B: Eukrohniidae; C: Spadellidae; D: Heterokrohniidae). For comparison purposes, *Pterosagitta draco* is depicted in bold with the Sagittidae (E) and with *Krohnitta subtilis* (F). Additional file 4:
**Dendrograms of morphometric similarity using full Procrustes distance (Riemannian shape distance ρ).** A: Primary hypothesis 1 (PH1): the anterior end of the posterior lateral fin in two-fin species is homologous to the posterior end of the unique lateral fin in one-fin species. B: Primary hypothesis 2 (PH2): the anterior end of the anterior lateral fin in two-fin species is homologous to the posterior end of the unique lateral fin in one-fin species. Surimposition of landmark points according to PH1 of all the studied chaetognaths are shown on Additional file 5. Additional file 5:
**Geometric morphometric method to explore body shape variations among Chaetognatha.** A: Landmark points (red dots) used for the morphometrics analysis according to the first hypothesis of primary homology (PH1: the anterior end of the posterior lateral fin in two-fin species is homologous to the anterior end of the unique lateral fin in one-fin species), here on *Mesosagitta minima* (drawing from Alvariño [38]). B: The landmarks, as input. C: The corrected landmarks, which straighten the specimen, according to their bilateral symmetry. The landmarks are: (1) Anterior end of the body; (2, 3) Anterior end of the trunk; (4, 5) Anterior end of the ventral nerve centre; (6, 7) Posterior end of the ventral nerve centre; (8, 9) Anterior end of the lateral fin; (10, 11) Posterior end of the lateral fin; (12, 13) Caudal septum; (14, 15) Anterior end of the seminal vesicle; (16, 17) Posterior end of the seminal vesicle; (18, 19) Anterior end of the caudal fin; (20) Posterior end of the body.
