# Supplementary material for: Evolutionary history of Chaetognatha inferred from molecular and morphological data: a case study for body plan simplification
Source: Front Zool. 2014 Nov 21;11:84. doi: 10.1186/s12983-014-0084-7 (PMC4254178; doi:10.1186/s12983-014-0084-7)
Supplement: Additional file 1: — Phylogeny of chaetognaths inferred from the SSU rRNA dataset 2 including both paralogy classes (138 sequences from 33 species: 80 class I and 58 class II sequences; 1679 base pairs long). Support values obtained using different reconstruction approaches are indicated at nodes in the following order: maximum Likelihood bootstrap probabilities (bv), approximative likelihood ratio test (aLRTv) and Bayesian posterior probabilities (pp). Support values are displayed when bv/aLRTv ≥75 or pp ≥0.85. Node absence in a given method is indicated by -. [file 12983_2014_84_MOESM1_ESM.pptx]

## Slide 1
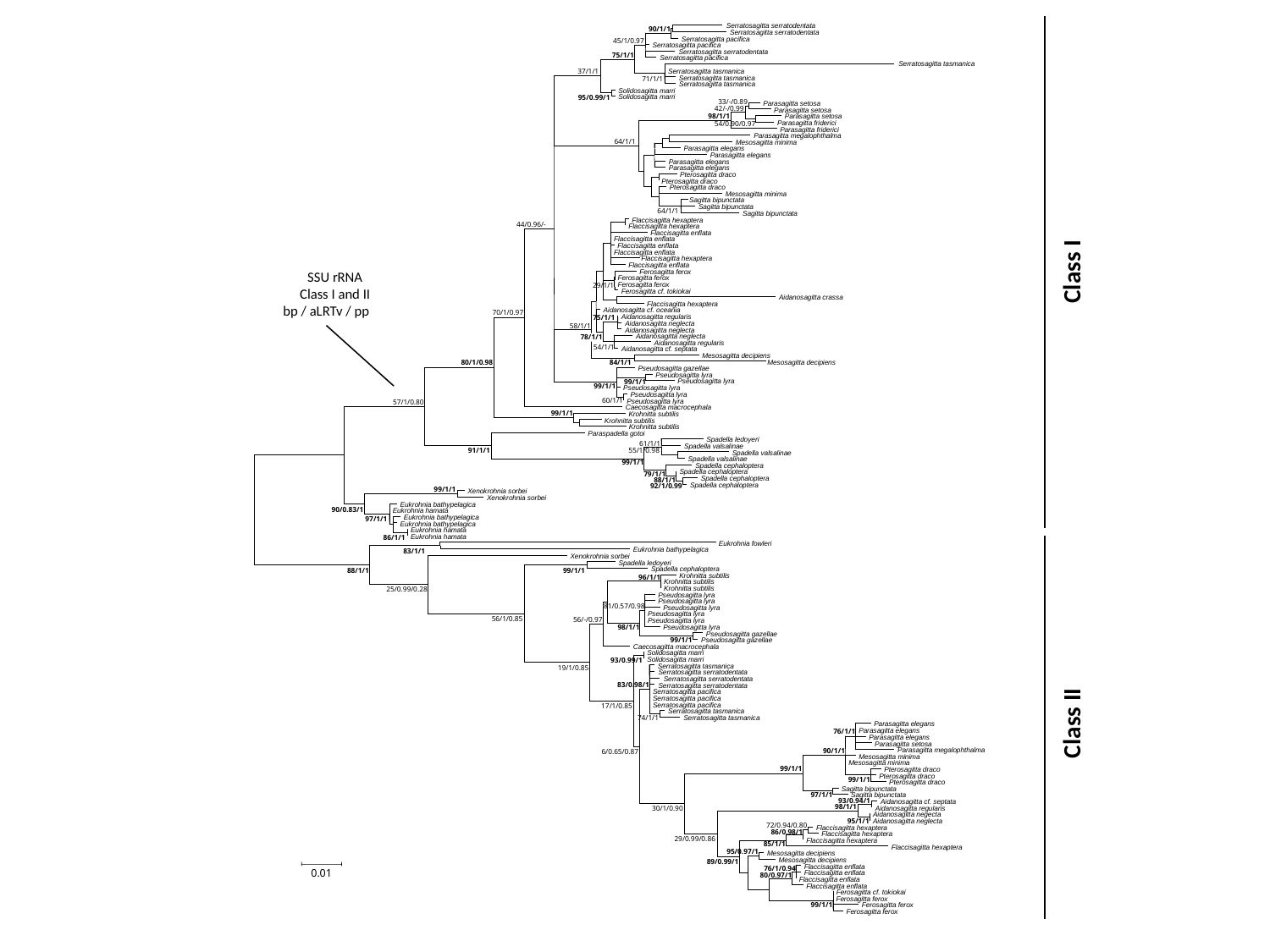

Serratosagitta serratodentata
90/1/1
 Serratosagitta serratodentata
 Serratosagitta pacifica
45/1/0.97
 Serratosagitta pacifica
 Serratosagitta serratodentata
75/1/1
 Serratosagitta pacifica
 Serratosagitta tasmanica
37/1/1
 Serratosagitta tasmanica
 Serratosagitta tasmanica
71/1/1
 Serratosagitta tasmanica
 Solidosagitta marri
 Solidosagitta marri
95/0.99/1
33/-/0.89
 Parasagitta setosa
42/-/0.99
 Parasagitta setosa
98/1/1
 Parasagitta setosa
 Parasagitta friderici
54/0.90/0.97
 Parasagitta friderici
 Parasagitta megalophthalma
64/1/1
 Mesosagitta minima
 Parasagitta elegans
 Parasagitta elegans
 Parasagitta elegans
 Parasagitta elegans
 Pterosagitta draco
 Pterosagitta draco
 Pterosagitta draco
 Mesosagitta minima
Sagitta bipunctata
 Sagitta bipunctata
64/1/1
 Sagitta bipunctata
 Flaccisagitta hexaptera
44/0.96/-
 Flaccisagitta hexaptera
 Flaccisagitta enflata
 Flaccisagitta enflata
 Flaccisagitta enflata
 Flaccisagitta enflata
Class I
Flaccisagitta hexaptera
 Flaccisagitta enflata
SSU rRNA
Class I and II
 Ferosagitta ferox
 Ferosagitta ferox
 Ferosagitta ferox
29/1/1
 Ferosagitta cf. tokiokai
 Aidanosagitta crassa
bp / aLRTv / pp
 Flaccisagitta hexaptera
 Aidanosagitta cf. oceania
70/1/0.97
 Aidanosagitta regularis
75/1/1
 Aidanosagitta neglecta
58/1/1
 Aidanosagitta neglecta
 Aidanosagitta neglecta
78/1/1
 Aidanosagitta regularis
54/1/1
 Aidanosagitta cf. septata
 Mesosagitta decipiens
84/1/1
Mesosagitta decipiens
80/1/0.98
 Pseudosagitta gazellae
 Pseudosagitta lyra
 Pseudosagitta lyra
99/1/1
99/1/1
 Pseudosagitta lyra
 Pseudosagitta lyra
60/1/1
 Pseudosagitta lyra
57/1/0.80
 Caecosagitta macrocephala
99/1/1
 Krohnitta subtilis
 Krohnitta subtilis
 Krohnitta subtilis
 Paraspadella gotoi
 Spadella ledoyeri
61/1/1
 Spadella valsalinae
55/1/0.98
91/1/1
 Spadella valsalinae
 Spadella valsalinae
99/1/1
 Spadella cephaloptera
 Spadella cephaloptera
79/1/1
 Spadella cephaloptera
88/1/1
 Spadella cephaloptera
92/1/0.99
99/1/1
 Xenokrohnia sorbei
 Xenokrohnia sorbei
 Eukrohnia bathypelagica
90/0.83/1
 Eukrohnia hamata
 Eukrohnia bathypelagica
97/1/1
 Eukrohnia bathypelagica
 Eukrohnia hamata
 Eukrohnia hamata
86/1/1
 Eukrohnia fowleri
 Eukrohnia bathypelagica
83/1/1
 Xenokrohnia sorbei
 Spadella ledoyeri
 Spadella cephaloptera
99/1/1
88/1/1
 Krohnitta subtilis
96/1/1
 Krohnitta subtilis
 Krohnitta subtilis
25/0.99/0.28
 Pseudosagitta lyra
 Pseudosagitta lyra
81/0.57/0.98
 Pseudosagitta lyra
 Pseudosagitta lyra
56/1/0.85
56/-/0.97
 Pseudosagitta lyra
98/1/1
 Pseudosagitta lyra
 Pseudosagitta gazellae
99/1/1
 Pseudosagitta gazellae
 Caecosagitta macrocephala
 Solidosagitta marri
 Solidosagitta marri
93/0.99/1
 Serratosagitta tasmanica
19/1/0.85
 Serratosagitta serratodentata
 Serratosagitta serratodentata
83/0.98/1
 Serratosagitta serratodentata
 Serratosagitta pacifica
 Serratosagitta pacifica
 Serratosagitta pacifica
17/1/0.85
Class II
 Serratosagitta tasmanica
 Serratosagitta tasmanica
74/1/1
 Parasagitta elegans
 Parasagitta elegans
76/1/1
 Parasagitta elegans
 Parasagitta setosa
 Parasagitta megalophthalma
90/1/1
6/0.65/0.87
 Mesosagitta minima
 Mesosagitta minima
99/1/1
 Pterosagitta draco
 Pterosagitta draco
99/1/1
 Pterosagitta draco
 Sagitta bipunctata
 Sagitta bipunctata
97/1/1
93/0.94/1
 Aidanosagitta cf. septata
98/1/1
 Aidanosagitta regularis
30/1/0.90
 Aidanosagitta negecta
 Aidanosagitta neglecta
95/1/1
72/0.94/0.80
 Flaccisagitta hexaptera
86/0.98/1
 Flaccisagitta hexaptera
29/0.99/0.86
 Flaccisagitta hexaptera
85/1/1
 Flaccisagitta hexaptera
95/0.97/1
 Mesosagitta decipiens
 Mesosagitta decipiens
89/0.99/1
 Flaccisagitta enflata
76/1/0.94
0.01
 Flaccisagitta enflata
80/0.97/1
 Flaccisagitta enflata
 Flaccisagitta enflata
 Ferosagitta cf. tokiokai
 Ferosagitta ferox
99/1/1
 Ferosagitta ferox
 Ferosagitta ferox
