# Supplementary material for: Evolutionary history of Chaetognatha inferred from molecular and morphological data: a case study for body plan simplification
Source: Front Zool. 2014 Nov 21;11:84. doi: 10.1186/s12983-014-0084-7 (PMC4254178; doi:10.1186/s12983-014-0084-7)
Supplement: Additional file 2: — Morphological data matrix. Unknown character states are indicated by NA and non-homologous characters are indicated by -. [file 12983_2014_84_MOESM2_ESM.docx]

## Additional file 2: Morphological data matrix.

Unknown character states are indicated by NA and non-homologous characters are indicated by -.

|  | C1 | C2 | C3 | C4 | C5 | C6 | C7 | C8 | C9 | C10 | C11 | C12 | C13 | C14 | C15 | C16 | C17 | C18 | C19 | C20 | C21 | C22 | C23 | C24 | C25 | C26 | C27 | C28 | C29 | C30 | C31 | C32 |
| --- | --- | --- | --- | --- | --- | --- | --- | --- | --- | --- | --- | --- | --- | --- | --- | --- | --- | --- | --- | --- | --- | --- | --- | --- | --- | --- | --- | --- | --- | --- | --- | --- |
| *Xenokrohnia sorbei* | 2 | 1 | - | 3 | 2 | 2 | 2 | NA | 1 | 1 | 1 | 1 | 5 | - | - | 2 | 1 | 1 | NA | 3 | 2 | 2 | 1 | 48 | 49 | 29.1 | 9 | 11 | 10 | 11 | 8 | 9 |
| *Eukrohnia bathypelagica* | 2 | 1 | - | 2 | 2 | 2 | 1 | NA | 1 | 1 | 1 | 1 | 3 | 2 | 1 | 1 | 1 | 1 | NA | 2 | 2 | 2 | 2 | 26 | 34 | 22.3 | 0 | 0 | 17 | 22 | 7 | 10 |
| *Eukrohnia hamata* | 2 | 1 | - | 2 | 2 | 2 | 1 | NA | 1 | 1 | 1 | 1 | 3 | 2 | 1 | 1 | 1 | 1 | NA | 2 | 2 | 2 | 2 | 19 | 24 | 20 | 0 | 0 | 23 | 25 | 8 | 9 |
| *Eukrohnia fowleri* | 2 | 1 | - | 2 | 2 | 2 | 1 | NA | 1 | 1 | 1 | 1 | 3 | 1 | 2 | 1 | 1 | 1 | NA | 2 | 2 | 2 | 2 | 22 | 25 | 20.6 | 0 | 0 | 28 | 30 | 10 | 13 |
| *Paraspadella gotoi* | 2 | 2 | - | 2 | 1 | 1 | 2 | 2 | 2 | 1 | 1 | 3 | 5 | 1 | 2 | 1 | 1 | 3 | 1 | 1 | 1 | 2 | 1 | 46 | 51 | 31.7 | 4 | 6 | 0 | 0 | 8 | 10 |
| *Spadella ledoyeri* | 2 | 2 | - | 2 | 1 | 1 | 2 | 2 | 1 | 1 | 1 | 6 | 2 | - | - | 1 | 1 | 1 | 1 | 3 | 2 | 2 | 1 | 50 | 53 | 29.1 | 3 | 5 | 3 | 3 | 10 | 11 |
| *Spadella cephaloptera* | 2 | 2 | - | 2 | 1 | 1 | 2 | 2 | 2 | 1 | 1 | 3 | 5 | 1 | 2 | 1 | 1 | 2 | 1 | 3 | 2 | 2 | 1 | 49 | 50 | 32 | 2 | 5 | 3 | 4 | 7 | 11 |
| *Spadella valsalinae* | 2 | 2 | - | 2 | 1 | 1 | 2 | 2 | 2 | 1 | 1 | 6 | 2 | 1 | 2 | 1 | 1 | 2 | 1 | 3 | 2 | 2 | 1 | 51 | 54 | 30.5 | 2 | 4 | 1 | 3 | 9 | 10 |
| *Krohnitta subtilis* | 2 | 3 | - | 1 | - | 2 | 1 | NA | 1 | 1 | 3 | 1 | 5 | 1 | 2 | 1 | 1 | 1 | 1 | 1 | 1 | 1 | 2 | 30 | 40 | 43.9 | 10 | 13 | 0 | 0 | 6 | 9 |
| *Caecosagitta macrocephala* | 2 | 4 | 1 | 1 | - | 2 | 1 | NA | 1 | 1 | 1 | 1 | 1 | 1 | 1 | 1 | 1 | 1 | NA | 3 | 1 | 2 | 2 | 32 | 35 | 29.6 | 6 | 10 | 20 | 28 | 10 | 12 |
| *Pterosagitta draco* | 2 | 2 | - | 1 | - | 2 | 1 | 1 | 1 | 1 | 1 | 2 | 3 | 1 | 2 | 1 | 1 | 1 | 1 | 3 | 1 | 2 | 1 | 38 | 45 | 29.4 | 10 | 10 | 18 | 18 | 10 | 10 |
| *Pseudosagitta gazellae* | 1 | 4 | 2 | 1 | - | 2 | 1 | NA | 2 | 1 | 1 | 3 | 1 | 1 | 2 | 1 | 2 | 1 | 4 | 3 | 1 | 2 | 2 | 10 | 18 | 20.4 | 3 | 9 | 5 | 11 | 7 | 14 |
| *Pseudosagitta lyra* | 1 | 4 | 2 | 1 | - | 2 | 1 | NA | 1 | 1 | 1 | 3 | 1 | 1 | 2 | 1 | 2 | 1 | 4 | 3 | 1 | 2 | 2 | 15 | 17 | 19.1 | 6 | 8 | 3 | 5 | 3 | 3 |
| *Flaccisagitta hexaptera* | 1 | 4 | 1 | 1 | - | 2 | 1 | 1 | 1 | 1 | 1 | 3 | 4 | 1 | 2 | 1 | 2 | 1 | 4 | 3 | 1 | 2 | 2 | 16 | 20 | 33.9 | 2 | 4 | 2 | 6 | 7 | 10 |
| *Flaccisagitta enflata* | 1 | 4 | 1 | 1 | - | 2 | 1 | 1 | 1 | 1 | 1 | 3 | 4 | 1 | 2 | 1 | 2 | 1 | 4 | 3 | 1 | 2 | 1 | 14 | 17 | 25 | 4 | 8 | 4 | 13 | 8 | 10 |
| *Serratosagitta serratodentata* | 2 | 4 | 1 | 1 | - | 2 | 1 | NA | 1 | 1 | 2 | 5 | 3 | 1 | 2 | 1 | 1 | 1 | 3 | 3 | 1 | 2 | 2 | 22 | 30 | 25.7 | 10 | 11 | 20 | 20 | 6 | 7 |
| *Serratosagitta pacifica* | 2 | 4 | 1 | 1 | - | 2 | 1 | NA | 1 | 1 | 2 | 5 | 3 | 1 | 2 | 1 | 1 | 1 | 3 | 3 | 1 | 2 | 1 | 22 | 28 | 26.5 | 7 | 13 | 16 | 25 | 4 | 7 |
| *Serratosagitta tasmanica* | 2 | 4 | 1 | 1 | - | 2 | 1 | NA | 1 | 1 | 2 | 5 | 3 | 1 | 2 | 1 | 1 | 1 | 3 | 3 | 1 | 2 | 2 | 20 | 30 | 22.9 | 2 | 9 | 3 | 19 | 6 | 8 |
| *Sagitta bipunctata* | 2 | 4 | 1 | 1 | - | 2 | 1 | 1 | 1 | 1 | 1 | 2 | 4 | 1 | 2 | 1 | 1 | 1 | 3 | 3 | 1 | 2 | 1 | 22 | 29 | 30.7 | 5 | 8 | 8 | 16 | 8 | 10 |
| *Mesosagitta minima* | 2 | 4 | 1 | 1 | - | 2 | 1 | NA | 2 | 2 | 1 | 2 | 4 | 1 | 2 | 1 | 1 | 1 | 2 | 3 | 1 | 2 | 2 | 17 | 21 | 24.1 | 3 | 5 | 6 | 12 | 7 | 9 |
| *Mesosagitta decipiens* | 2 | 4 | 1 | 1 | - | 2 | 1 | NA | 2 | 2 | 1 | 2 | 4 | 1 | 2 | 1 | 1 | 1 | 2 | 3 | 1 | 2 | 2 | 25 | 31 | 21.4 | 8 | 10 | 19 | 22 | 5 | 8 |
| *Parasagitta elegans* | 2 | 4 | 1 | 1 | - | 2 | 1 | 1 | 2 | 2 | 1 | 3 | 4 | 1 | 2 | 1 | 1 | 1 | 3 | 3 | 1 | 2 | 1 | 17 | 25 | 21.5 | 2 | 8 | 12 | 18 | 9 | 11 |
| *Parasagitta megalophthalma* | 2 | 4 | 1 | 1 | - | 2 | 1 | NA | 2 | 2 | 1 | 2 | 4 | 1 | 2 | 1 | 1 | 1 | 3 | 3 | 1 | 2 | 1 | 25 | 31 | 27.6 | 10 | 10 | 18 | 18 | 7 | 8 |
| *Parasagitta setosa* | 2 | 4 | 1 | 1 | - | 2 | 1 | 1 | 1 | 1 | 1 | 2 | 5 | 1 | 2 | 1 | 1 | 1 | 3 | 3 | 1 | 2 | 1 | 16 | 25 | 30.5 | 8 | 8 | 16 | 16 | 8 | 9 |
| *Parasagitta friderici* | 2 | 4 | 1 | 1 | - | 2 | 1 | NA | 1 | 1 | 1 | 2 | 5 | 1 | 2 | 1 | 1 | 1 | 3 | 3 | 1 | 2 | 1 | 25 | 28 | 27.2 | 3 | 8 | 6 | 17 | 5 | 9 |
| *Solidosagitta zetesios* | 2 | 4 | 1 | 1 | - | 2 | 1 | NA | 2 | 2 | 1 | 1 | 1 | 1 | 2 | 1 | 2 | 1 | 2 | 3 | 1 | 2 | 2 | 20 | 23 | 30.1 | 8 | 12 | 15 | 22 | 8 | 11 |
| *Solidosagitta marri* | 2 | 4 | 1 | 1 | - | 2 | 1 | NA | 2 | 2 | 1 | 1 | 2 | 1 | 2 | 1 | 1 | 1 | 2 | 3 | 1 | 2 | 1 | 20 | 28 | 32.7 | 6 | 8 | 14 | 17 | 7 | 11 |
| *Aidanosagitta oceania* | 2 | 4 | 1 | 1 | - | 2 | 1 | NA | 2 | 1 | 1 | 4 | 3 | 1 | 2 | 1 | 1 | 1 | 2 | 3 | 1 | 2 | 1 | 25 | 29 | 26.3 | 5 | 8 | 15 | 20 | 6 | 7 |
| *Aidanosagitta crassa* | 1 | 4 | 1 | 1 | - | 2 | 1 | NA | 2 | 1 | 1 | 4 | 3 | 1 | 2 | 1 | 1 | 1 | 2 | 3 | 1 | 2 | 1 | 28 | 33 | 27 | 7 | 11 | 18 | 24 | 8 | 10 |
| *Aidanosagitta neglecta* | 2 | 4 | 1 | 1 | - | 2 | 1 | NA | 2 | 1 | 1 | 4 | 3 | 1 | 2 | 1 | 1 | 1 | 2 | 3 | 1 | 2 | 1 | 26 | 30 | 30.9 | 7 | 7 | 18 | 18 | 6 | 7 |
| *Aidanosagitta regularis* | 2 | 4 | 1 | 1 | - | 2 | 1 | NA | 2 | 1 | 1 | 4 | 3 | 1 | 2 | 1 | 1 | 1 | 2 | 3 | 1 | 2 | 1 | 32 | 33 | 26.7 | 3 | 4 | 6 | 6 | 11 | 11 |
| *Aidanosagitta septata* | 2 | 4 | 1 | 1 | - | 2 | 1 | NA | 2 | 1 | 1 | 4 | 3 | 1 | 2 | 1 | 1 | 1 | 2 | 3 | 1 | 2 | 1 | 27 | 29 | 29.4 | 4 | 5 | 8 | 14 | 6 | 8 |
| *Ferosagitta ferox* | 2 | 4 | 1 | 1 | - | 2 | 1 | NA | 2 | 1 | 1 | 4 | 5 | 1 | 2 | 1 | 1 | 1 | 3 | 3 | 1 | 2 | 1 | 25 | 31 | 21.8 | 7 | 10 | 12 | 14 | 5 | 6 |
| *Ferosagitta tokiokai* | 2 | 4 | 1 | 1 | - | 2 | 1 | NA | 2 | 1 | 1 | 4 | 5 | 1 | 2 | 1 | 1 | 1 | 3 | 3 | 1 | 2 | 1 | 22 | 25 | 25 | 6 | 9 | 12 | 14 | 6 | 7 |
