# Supplementary material for: Evolutionary history of Chaetognatha inferred from molecular and morphological data: a case study for body plan simplification
Source: Front Zool. 2014 Nov 21;11:84. doi: 10.1186/s12983-014-0084-7 (PMC4254178; doi:10.1186/s12983-014-0084-7)
Supplement: Additional file 3: — Diagrammatic representation of morphometric measures used for the analysis of body shape similarities. A: Superimposition of all the studied chaetognaths, altering the scales, to superimpose their anterior and posterior ends (points 1 and 20). B-F: same graphs family per family (B: Eukrohniidae; C: Spadellidae; D: Heterokrohniidae). For comparison purposes, Pterosagitta draco is depicted in bold with the Sagittidae (E) and with Krohnitta subtilis (F). [file 12983_2014_84_MOESM3_ESM.pdf]

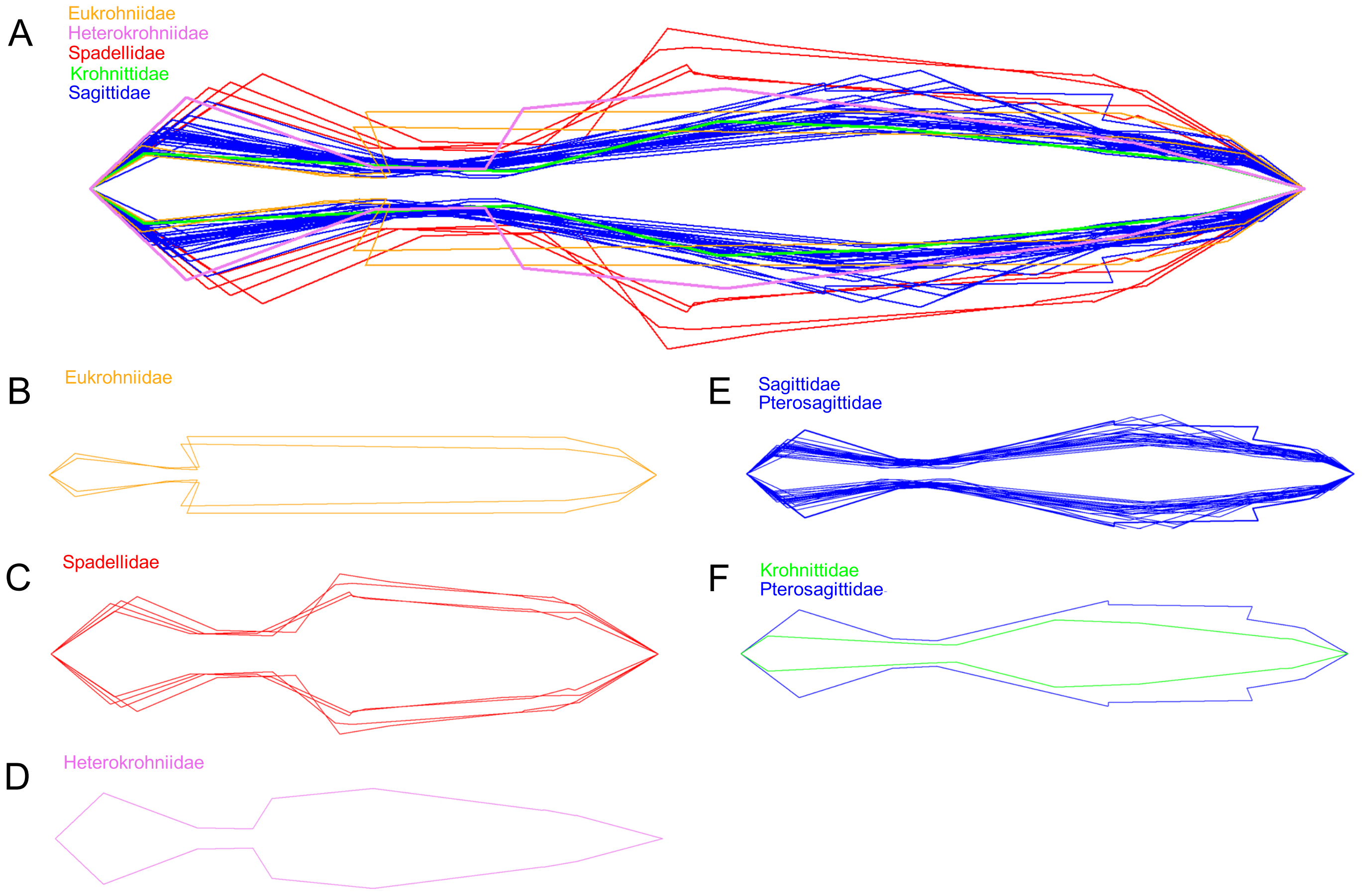
A
Eukrohniidae
Heterokrohniidae
Spadellidae
Krohnittidae
Sagittidae
B
Eukrohniidae
C
Spadellidae
D
Heterokrohniidae
E
Sagittidae
Pterosagittidae
F
Krohnittidae
Pterosagittidae
