# Supplementary material for: Evolutionary history of Chaetognatha inferred from molecular and morphological data: a case study for body plan simplification
Source: Front Zool. 2014 Nov 21;11:84. doi: 10.1186/s12983-014-0084-7 (PMC4254178; doi:10.1186/s12983-014-0084-7)
Supplement: Additional file 4: — Dendrograms of morphometric similarity using full Procrustes distance (Riemannian shape distance ρ). A: Primary hypothesis 1 (PH1): the anterior end of the posterior lateral fin in two-fin species is homologous to the posterior end of the unique lateral fin in one-fin species. B: Primary hypothesis 2 (PH2): the anterior end of the anterior lateral fin in two-fin species is homologous to the posterior end of the unique lateral fin in one-fin species. Surimposition of landmark points according to PH1 of all the studied chaetognaths are shown on Additional file 5. [file 12983_2014_84_MOESM4_ESM.pptx]

## Slide 1
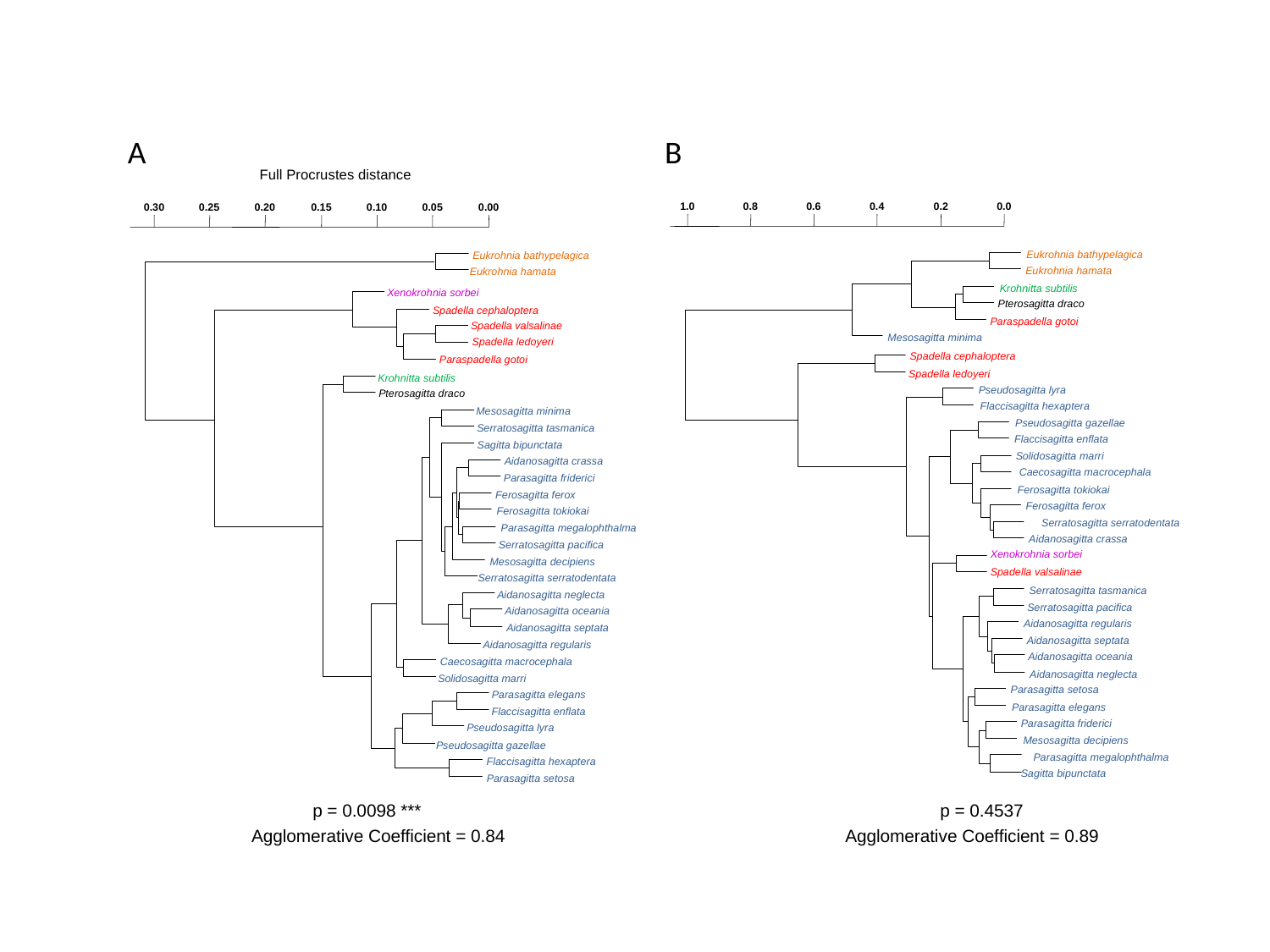

A
B
Full Procrustes distance
0.4
0.2
0.0
1.0
0.8
0.6
0.10
0.05
0.00
0.20
0.15
0.30
0.25
Eukrohnia bathypelagica
Eukrohnia hamata
Krohnitta subtilis
Pterosagitta draco
Paraspadella gotoi
Mesosagitta minima
Spadella cephaloptera
Spadella ledoyeri
Pseudosagitta lyra
Flaccisagitta hexaptera
Pseudosagitta gazellae
Flaccisagitta enflata
Solidosagitta marri
Caecosagitta macrocephala
Ferosagitta tokiokai
Ferosagitta ferox
Serratosagitta serratodentata
Aidanosagitta crassa
Xenokrohnia sorbei
Spadella valsalinae
Serratosagitta tasmanica
Serratosagitta pacifica
Aidanosagitta regularis
Aidanosagitta septata
Aidanosagitta oceania
Aidanosagitta neglecta
Parasagitta setosa
Parasagitta elegans
Parasagitta friderici
Mesosagitta decipiens
Parasagitta megalophthalma
Sagitta bipunctata
Eukrohnia bathypelagica
Eukrohnia hamata
Xenokrohnia sorbei
Spadella cephaloptera
Spadella valsalinae
Spadella ledoyeri
Paraspadella gotoi
Krohnitta subtilis
Pterosagitta draco
Mesosagitta minima
Serratosagitta tasmanica
Sagitta bipunctata
Aidanosagitta crassa
Parasagitta friderici
Ferosagitta ferox
Ferosagitta tokiokai
Parasagitta megalophthalma
Serratosagitta pacifica
Mesosagitta decipiens
Serratosagitta serratodentata
Aidanosagitta neglecta
Aidanosagitta oceania
Aidanosagitta septata
Aidanosagitta regularis
Caecosagitta macrocephala
Solidosagitta marri
Parasagitta elegans
Flaccisagitta enflata
Pseudosagitta lyra
Pseudosagitta gazellae
Flaccisagitta hexaptera
Parasagitta setosa
p = 0.0098 ***
p = 0.4537
Agglomerative Coefficient = 0.84
Agglomerative Coefficient = 0.89
