# Supplementary figures and images for: Evolutionary history of Chaetognatha inferred from molecular and morphological data: a case study for body plan simplification
Source: Front Zool. 2014 Nov 21;11:84. doi: 10.1186/s12983-014-0084-7 (PMC4254178; doi:10.1186/s12983-014-0084-7)

**A**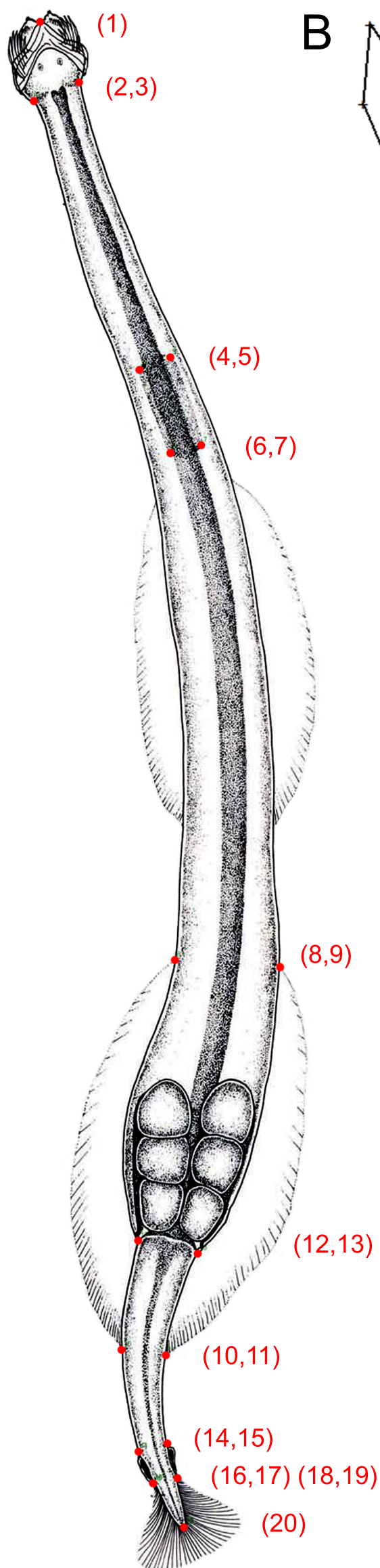**B**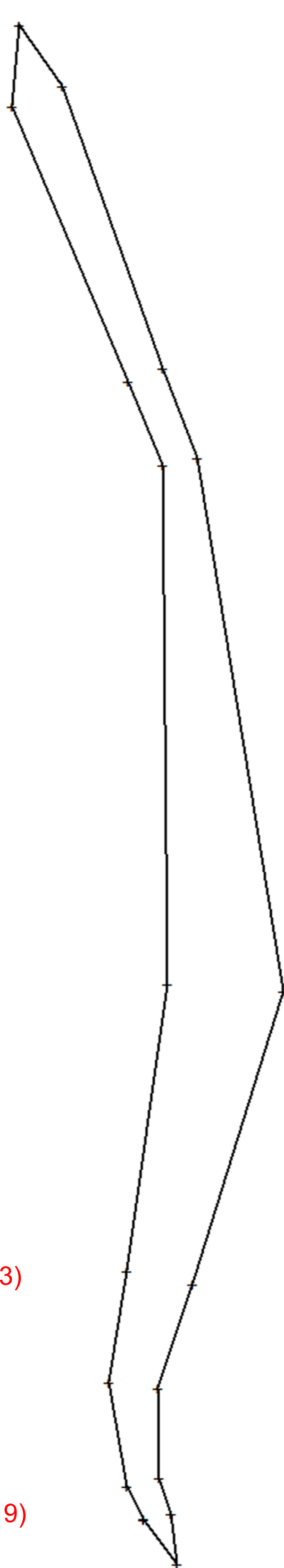**C**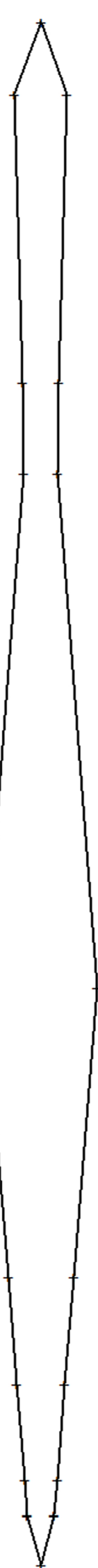

Supplement: Additional file 5: — Geometric morphometric method to explore body shape variations among Chaetognatha. A: Landmark points (red dots) used for the morphometrics analysis according to the first hypothesis of primary homology (PH1: the anterior end of the posterior lateral fin in two-fin species is homologous to the anterior end of the unique lateral fin in one-fin species), here on Mesosagitta minima (drawing from Alvariño [38]). B: The landmarks, as input. C: The corrected landmarks, which straighten the specimen, according to their bilateral symmetry. The landmarks are: (1) Anterior end of the body; (2, 3) Anterior end of the trunk; (4, 5) Anterior end of the ventral nerve centre; (6, 7) Posterior end of the ventral nerve centre; (8, 9) Anterior end of the lateral fin; (10, 11) Posterior end of the lateral fin; (12, 13) Caudal septum; (14, 15) Anterior end of the seminal vesicle; (16, 17) Posterior end of the seminal vesicle; (18, 19) Anterior end of the caudal fin; (20) Posterior end of the body. [file 12983_2014_84_MOESM5_ESM.pdf]
